# Identification of lignin genes and regulatory sequences involved in secondary cell wall formation in *Acacia auriculiformis *and *Acacia mangium *via *de novo *transcriptome sequencing

**DOI:** 10.1186/1471-2164-12-342

**Published:** 2011-07-05

**Authors:** Melissa ML Wong, Charles H Cannon, Ratnam Wickneswari

**Affiliations:** 1 School of Environmental and Natural Resource Sciences, Faculty of Science and Technology, Universiti Kebangsaan Malaysia, UKM Bangi 43600, Selangor, Malaysia; 2 Ecological Evolution Group, Xishuangbanna Tropical Botanical Garden, Chinese Academy of Science, Menglun, Mengla 666303, Yunnan, P. R. China; 3 Department of Biological Sciences, Texas Tech University, Lubbock, TX 79409 USA

## Abstract

**Background:**

*Acacia auriculiformis *× *Acacia mangium *hybrids are commercially important trees for the timber and pulp industry in Southeast Asia. Increasing pulp yield while reducing pulping costs are major objectives of tree breeding programs. The general monolignol biosynthesis and secondary cell wall formation pathways are well-characterized but genes in these pathways are poorly characterized in *Acacia *hybrids. RNA-seq on short-read platforms is a rapid approach for obtaining comprehensive transcriptomic data and to discover informative sequence variants.

**Results:**

We sequenced transcriptomes of *A. auriculiformis *and *A. mangium *from non-normalized cDNA libraries synthesized from pooled young stem and inner bark tissues using paired-end libraries and a single lane of an Illumina GAII machine. *De novo *assembly produced a total of 42,217 and 35,759 contigs with an average length of 496 bp and 498 bp for *A. auriculiformis *and *A. mangium *respectively. The assemblies of *A. auriculiformis *and *A. mangium *had a total length of 21,022,649 bp and 17,838,260 bp, respectively, with the largest contig 15,262 bp long. We detected all ten monolignol biosynthetic genes using Blastx and further analysis revealed 18 lignin isoforms for each species. We also identified five contigs homologous to R2R3-MYB proteins in other plant species that are involved in transcriptional regulation of secondary cell wall formation and lignin deposition. We searched the contigs against public microRNA database and predicted the stem-loop structures of six highly conserved microRNA families (miR319, miR396, miR160, miR172, miR162 and miR168) and one legume-specific family (miR2086). Three microRNA target genes were predicted to be involved in wood formation and flavonoid biosynthesis. By using the assemblies as a reference, we discovered 16,648 and 9,335 high quality putative Single Nucleotide Polymorphisms (SNPs) in the transcriptomes of *A. auriculiformis *and *A. mangium*, respectively, thus yielding useful markers for population genetics studies and marker-assisted selection.

**Conclusion:**

We have produced the first comprehensive transcriptome-wide analysis in *A. auriculiformis *and *A. mangium *using *de novo *assembly techniques. Our high quality and comprehensive assemblies allowed the identification of many genes in the lignin biosynthesis and secondary cell wall formation in *Acacia *hybrids. Our results demonstrated that Next Generation Sequencing is a cost-effective method for gene discovery, identification of regulatory sequences, and informative markers in a non-model plant.

## Background

Next Generation Sequencing (NGS) is quickly becoming the standard for the generation of cheap, accurate and high throughput DNA sequence data [1]. The major NGS platforms are Roche 454 GS-FLX Titanium (330 bp), Illumina GAIIx (75-100 bp) and SOLiD3 (50 bp), which differ in read length, error rate and cost [2]. Transcriptome sequencing using NGS, commonly known as RNA-Seq, enables rapid and cost-effective gene and marker discovery, gene expression analysis, detection of rare variants and splice isoforms. Most previous studies have involved sequencing plant transcriptomes with completed reference genomes available, such as *Arabidopsis thalina *[3,4], *Medicago truncatula *[5] and *Zea mays *[6,7]. Direct sequencing of the transcriptome of non-model organisms has the potential to rapidly generate valuable genomic resources in poorly known species. However, *de novo *transcriptome assembly is challenging due to short reads, lack of reference sequences and the need for development of improved bioinformatic tools to facilitate data analysis [8].

Most *de novo *transcriptome studies have used the Roche 454 platforms [9-13] as the longer reads allow more reliable *de novo *assembly, however, the reactions are relatively expensive, reducing the potential sequencing coverage which plays a major role in the accuracy of *de novo *assembly. Hybrid sequencing approaches using 454/Illumina technologies can successfully reduce cost and compensate for different sequencing technology biases [14,15]. While sequencing exclusively using Illumina technology, the most widely published NGS platform is an attractive and cheap alternative as the high coverage obtained can overcome sequencing error rates and short read length, relatively few *de novo *transcriptome studies have exploited these advantages in plants [16]https://atgc-illumina.googlecode.com/files/PAG_2010_AKozik_V09.pdf. As read lengths increase, paired-end library construction techniques improve and costs continue to go down, Illumina RNA-seq will become a powerful tool for transcriptome characterization of non-model plants.

*Acacia mangium *and *Acacia auriculiformis *are important forest tree species, belonging to the Fabaceae or Legume family, and are native to Australia, Papua New Guinea and Indonesia. *A. mangium *is widely planted in Southeast Asia because of its superior growth, wide site suitability and multiple uses [17,18] while *A. auriculiformis *has higher adaptability, greater durability and is less susceptible to diseases than *A. mangium*. *A. auriculiformis *and *A. mangium *are predominantly out-crossing [19,20]. Naturally-crossed *Acacia *hybrids were first noted in Sabah in the late 1970s [21]. These hybrids possessed many attractive traits highly sought in tree improvement, such as enhanced growth, form, disease resistance and adaptability. For the wood and pulp industry, the *Acacia *hybrids have great potential as raw material due to superior growth, longer wood fibers and better pulp quality over their parents [22]. Low lignin and high cellulose content are desirable in the pulping process and studies have shown increased accumulation of cellulose occurs when lignin is reduced in plants [23]. The monolignol biosynthesis pathway is well-characterized but the coordination and regulation of genes in the pathway is not well-understood. Recent studies revealed that known regulatory sequences, including several classes of transcription factors and microRNAs play important roles in regulation of lignin and wood formation [24,25]. These regulatory sequences may be good candidates in selective breeding and genetic engineering programs to increase pulp yield and reduce pulping costs.

The C-value for *A. auriculiformis *and *A. mangium *(both 2n = 26) are estimated to be 0.83 pg and 0.65 pg respectively [26] while *A. auriculiformis *× *A. mangium *hybrid genome size is estimated to be 750 Mb [27], making the hybrid genome 1.4 times larger than the *Populus trichocarpa *genome. Currently, no genome sequences for any *Acacia *species are available although the genomes of several model legume species like *M. trunc*atula and *Glycine max *have been sequenced. Unfortunately, all of these model legumes are in a separate subfamily, the Faboideae, while *Acacia *species are in the Mimosoideae subfamily. In terms of EST resources for *A. mangium*, a total of 147 from floral tissues [28], 8,963 from secondary xylem and shoot tissue [29] and 2,459 from inner bark of the *A. auriculiformis *× *A. mangium *hybrid [30] have been deposited in the NCBI dbEST. However, no genomic resources is available for *A. auriculiformis*. Several important genes involved in monolignol biosynthesis and wood-related pathways including *cinammate 4-hydroxylase *(C4H), *caffeoyl CoA 3-O-methyltransferase *(CCoAOMT), *cinnamyl alcohol dehydrogenase *(CAD), *phenylalanine ammonia lyase *(PAL), *caffeic acid O-methyltransferase *(COMT) and *cellulose synthase *(CesA) have been successfully isolated and characterized from the *Acacia *hybrid [30,31].

Conventional breeding programs for the improvement of forest trees are slow, laborious and land intensive due to the long life cycle and large size of trees. The application of genomic approaches facilitated by emerging DNA sequencing technologies may significantly accelerate the breeding program. Due to the lack of genomic resources for tree crops particularly tropical species, the simple discovery of genes controlling wood-related traits will be a major step forward. Ultimately, the development of large-scale genomic resources will facilitate the application of linkage and association mapping within tree improvement programs.

Here we applied paired-end Illumina GAII sequencing to non-normalized cDNAs of *A. auriculiformis *and *A. mangium *to discover important genes involved in lignin and secondary cell wall formation in these non-model tree species. Using standard *de novo *assembly algorithms, we examined the quality of the contigs generated and attempted to identify wood-related genes particularly genes and their isoforms in the monolignol biosynthesis pathway. We also sought to identify potential transcription factors involved in secondary wood formation and lignin deposition, and highly conserved microRNAs and their wood-related gene targets. A major objective in our analysis was to detect a large number of informative SNPs to be used for linkage mapping of hybrid progenies and population genetic studies of the two parental species. Our results could provide powerful tools for the efficient selection of hybrid offsprings with favorable traits, allowing rapid and continued improvement.

## Results and Discussion

### De novo transcriptome assembly

In this study, we constructed non-normalized cDNA libraries for each parental species as this will produce more full length transcripts for significant gene discovery. Each library was sequenced using one lane of a flow cell on the Illumina GAII platform using paired end protocols. We obtained 19,899,637 and 17,859,793 51 bp paired-end raw reads for *A. auriculiformis *and *A. mangium*, respectively. Filtering and conversion to FASTQ format resulted in 13,648,154 and 12,621,865 paired-end reads for *A. auriculiformis *and *A. mangium *respectively. After filtering of ribosomal RNA sequences, 51-57% of the reads remained with an average Phred score of 34 - 35.

**Figure 1:**
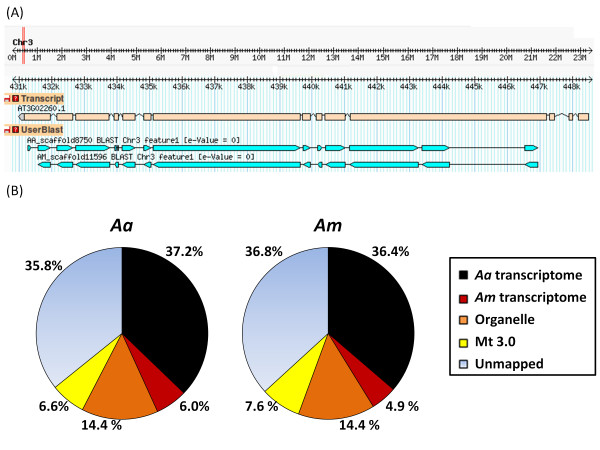
***De novo *transcriptome assemblies**. A) Alignment of the longest contig to the *Arabidopsis thaliana *genome. Blastn alignment of the longest contig of *A. auriculiformis *and *A. mangium *to *Arabidopsis thaliana *BIG/ubiquitin ligase gene located at chromosome 3 (E-value = 0) as showed in GBrowse; B) Proportion of filtered reads mapped to *Acacia *transcriptomes and other genomes.

The filtered reads were used to perform *de novo *assembly using a number of software such as Velvet [32], SOAPdenovo [33] and Oases [34], however, we found SOAPdenovo produced the longest assemblies despite using longer k-mers. We assessed different k-mer sizes and chose 29-mer to obtain a good tradeoff between assembly size and accuracy. *De novo *transcriptome assembly for *A. auriculiformis *(subsequently referred to as '*Aa*') and *A. mangium *(subsequently referred to as '*Am*') produced 42,217 and 35,759 contigs with an N50 contig size of 948 bp and 938 bp, a longest contig of 15,262 bp and 15,220 bp, and an average length of 496 bp and 498 bp respectively (Table 1). The sequencing depth was estimated to be 18.7 × and 18.3 × respectively. Blastx indicated that the longest contig of *Aa *and *Am *were homologs of the *A. thaliana *BIG; binding/ubiquitin-protein ligase/zinc ion binding gene (Figure 1A). This gene which is one of the longest genes in plants, was also reported in *de novo *transcriptome assembly of lettuce https://atgc-illumina.googlecode.com/files/PAG_2010_AKozik_V09.pdf.

**Table 1:** Summary of *de novo *transcriptome assembly

Species	*A. auriculiformis*	*A. mangium*
Filtered reads (paired-ends)	7,743,336	6,392,887
Filtered reads (single-ends)	15,486,672	12,785,774
Total assembled size (bp)	21,022,649	17,838,260
Number of contigs and scaffolds	42,217	35,759
Longest contig (bp)	15,262	15,220
N50 (bp)	949	938
Average length (bp)	498	496
GC content (%)	43	43
Estimated coverage	18.7 ×	18.3 ×

To determine the similarity at the nucleotide level between the transcriptomes, we first mapped filtered reads to their corresponding *de novo *contigs before mapping each set of reads against the contigs obtained from the other species. To substantially increase the number of mappable reads, we mapped single-end reads using Bowtie -v setting allowing three mismatches. A total of 5,766,757 *Aa *single-end reads (37.24%) and 4,647,280 *Am *single-end reads (36.35%) mapped to their corresponding contigs. We observed only a small drop (roughly 15%) in the proportion of mappable reads from one *Acacia *species to the contigs of the other *Acacia *species indicating that the two transcriptomes shared a great deal of identity at the nucleotide level and are closely related.

The observation that a large proportion of filtered reads failed to map to the *Acacia *transcriptomes (> 60%) led us to investigate their origins by mapping to various genomes (Figure 1B). A further 5-6% of the reads mapped to the transcriptome of the other *Acacia *species probably due to differentially expressed transcripts. We discovered that approximately 14% of the reads mapped to mitochondrial and chloroplast genomes of *A. thaliana*, suggesting a significant amount of mitochondrial and chloroplast transcripts were sequenced. We suspect that mitochondrion sequences may not be assembled due to the highly heterozygous nature of genomes that were present in high copy number. We tried to map the remaining reads to several model plant genomes but found less than 10% mappable reads and no huge differences between these plant genomes. The number of reads mappable to the model legume, *M. truncatula *masked genome version Mt3.0 was 6.6-7.6%. The remaining ~36% of filtered reads were unmappable possibly due to several reasons. Some of these reads may be unique *Acacia *sequences from intergenic and intronic regions based on observation from Wang et al. [35] study that reported 40.75% of RNA-Seq reads from *Aspergillus oryzae *were located at these regions. Other reasons such as lack of *Acacia *genome information, poor quality reads and microbial contamination may have contributed to the large number of unmappable reads.

### Discovery of monolignol biosynthetic genes and isoforms

The monolignol biosynthesis pathway consists of several large protein families with members commonly known as isoforms. Isoform identification is challenging due to presence of many closely related superfamily members ("like") in the transcriptome, i.e. 27 "like" proteins of COMT, CCR and 4CL were observed in *A. thaliana *[36]. In this study, we found a total of 52 contigs in *Aa *and *Am *transcriptomes with E-value ≤ 1E-10 corresponding to all ten monolignol genes in *A. thaliana*. Gene identification using Blast alone often resulted in an overestimation of the total number of genes and isoforms. Shi et al. [37] reported 95 members of phenylpropanoid genes found in *P. trichocarpa *genome using Blastp (E-value ≤ 1E-3), however, many are proposed to be unrelated to monolignol biosynthesis pathway based on phylogenetic and expression analysis. Therefore, we tried to remove unrelated proteins by checking the conserved motifs which provide important clues in protein function and identity. We excluded contigs with low homology to *A. thaliana *monolignol genes (less than 55% identity) and we checked the remaining contigs for conserved amino acid motifs identified in previous studies [38-46] from the protein alignments (Additional File 1).

**Figure 2:**
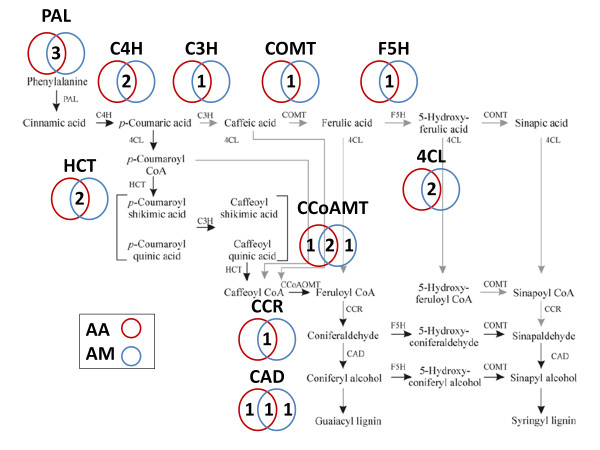
**Monolignol biosynthesis pathway isoforms of *A. auriculiformis *and *A. mangium***. The number of isoforms found in *A. auriculiformis *(showed in red circle) and *A. mangium *(showed in blue circle) based on Blastx (E-value ≤ 1E-10) and conserved motifs. The number of orthologous isoforms shared by both species is indicated in the overlapping region. The figure is reprinted with permission from The Brazilian Society of Genetics. *Phenylalanine ammonia lyase *(PAL), *cinammate 4-hydroxylase *(C4H), *4-coumarate 3-hydroxylase *(C3H), *caffeic acid O-methyltransferase*(COMT), *ferulate 5-hydroxylase (F5H), 4-coumarate:CoA ligase* (4CL), *hydroxycinnamoyl-CoA shikimate/quinatehydroxy-cinnamoyltransferase *(HCT), *caffeoyl CoA 3-O-methyltransferase *(CCoAOMT), *cinnamyl alcohol dehydrogenase *(CAD), *cinnamoyl Co-A reductase *(CCR).

We were able to detect all ten genes involved in monolignol biosynthesis pathway, namely *phenylalanine ammonia lyase *(PAL), *cinammate 4-hydroxylase *(C4H), *4-coumarate 3-hydroxylase *(C3H), *caffeic acid O-methyltransferase *(COMT), *ferulate 5-hydroxylase *(F5H), *4-coumarate:CoA ligase *(4CL), *hydroxycinnamoyl-CoA shikimate/quinatehydroxy-cinnamoyltransferase *(HCT), *caffeoyl CoA 3-O-methyltransferase *(CCoAOMT), *cinnamyl alcohol dehydrogenase *(CAD), *cinnamoyl Co-A reductase *(CCR) compared to traditional EST sequencing in *A. mangium *[29] and *A. auriculiformis *× *A. mangium *hybrid [30]. We discovered more than one isoform for half of the genes which failed to be detected by EST sequencing. We identified a total of 18 isoforms for each species whereas 16 orthologous isoforms were shared in both species (Figure 2). All isoforms shared high identities with the corresponding *A. thaliana *genes where C3H shared the highest identity (68-85%), followed by PAL (71-84%), C4H (64-84%), CCoAOMT (64-83%), HCT (72-78%), CAD (58-76%), COMT (74%), CCR (73%), 4CL (57-71%) and F5H (59-62%). Our observations that orthologous isoforms of *Aa *and *Am *shared at least 99% similarity at both nucleotide and protein level while isoforms within the same family usually do not share an exact match of more than 16 nucleotides are important in determining the number of isoforms for both species.

The total assembled sequence lengths of the 36 isoforms ranged from 503 to 2,460 bp and only 14 contained complete open reading frame (ORF). No polyadenylation site was observed as expected because short polyA sequences failed to be assembled. One limitation of our sequence analysis is the presence of gap region in the contigs. Half of the assembled sequences contain gap regions with the total size range of 13 - 403 bp. These regions which were masked by Ns often occur at low coverage area where two contigs or mate pairs are connected during scaffolding. Although most *de novo *assemblers can estimate the size of the gap region, the predicted size is not always correct and sometimes resulting in inaccurate protein prediction. It is recommended to double-check the protein sequences by translating each fragment in gapped assemblies using other protein prediction software. Missing data poses a challenge to sequence comparison and analysis and therefore, gap filling by resequencing should be done in the future.

The total number of isoforms detected in this study is generally lower than those found in *A. thaliana *[36] and *P. trichocarpa *[37]. The identified isoforms possessed 99% DNA sequence identity with previously characterized isoforms from *A. auriculiformis *× *A. mangium hybrid *for the five isoforms that we examined, namely PAL, C4H, COMT, CCoAOMT, and CAD [31]. The high sequence similarity between of *A. auriculiformis*, *A. mangium *and their hybrids will allow more efficient cross amplification in gene isolation and characterization efforts. Given that several isoforms were only found in one species, greater sequencing depth is required for our analysis to overcome incomplete assemblies and sampling biases, previously observed in genomic sequences of *Pseudomonas syringae *strains [47]. Nevertheless, transcriptome sequencing of other tissues such as secondary xylem will provide more differentially expressed isoforms which can be new targets for the improvement of wood properties.

### Identification of wood-related transcription factors

**Figure 3:**
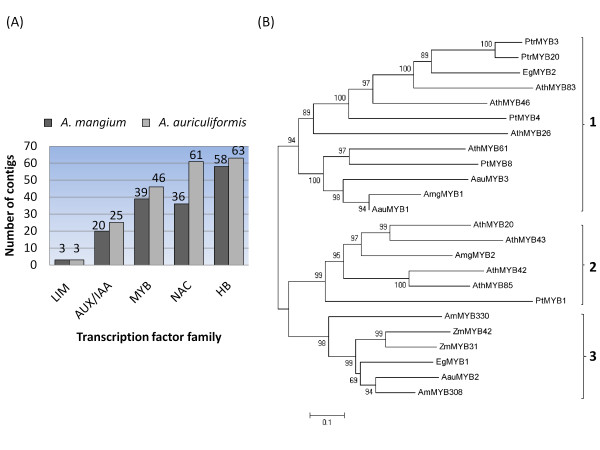
**Transcription factors involved in regulation of wood formation and lignin biosynthesis**. (A) Number of contigs found in *A. auriculiformis *and *A. mangium *corresponding to five major wood-related transcription factor families (E-value ≤ 1E-10); (B) Phylogenetic tree of R2R3-MYBs of *A. auriculiformis*, *A. mangium *and other plant species that are involved in secondary cell wall formation. The neighbour-joining tree was obtained with MEGA 5.0 software and ClustalW2 alignments of full length amino acid sequences. Bootstrap values > 50% are shown. The bar indicates an evolutionary distance of 0.1. Ath: *Arabidopsis thaliana*, Am: *Anthirinum majus*, Zm: *Zea mays*, Pt: *Pinus taeda*, Ptr: *Populus trichocarpa*, Eg: *Eucalyptus gunnii*, Aau: *A. auriculiformis*, Amg: *A. mangium*.

We found 1,306 *Aa *and 1,160 *Am *contigs with high sequence identity (E-value ≤ 1E-10) corresponding to 72 and 73 families out of 82 *A. thaliana *transcription factor families downloaded from PlnTFDB [48]. The five most abundant transcriptional gene families were WKRY, Orphans, PHD, HB and the MYB-related group. Several major classes of transcription factors involved in lignin and wood formation were found in both species (Figure 3A), generally in similar numbers of contigs, although the NAC family was substantially more abundant in *Aa*. Additionally, eight *Aa *and nine *Am *contigs were identified as class III HD-ZIP, a member of Homeobox (HB) family.

Some members of the R2R3-MYB family are known to be involved in controlling lignin deposition and secondary wall formation by interacting with other R2R3-MYB genes, activated by NAC transcription factor master switches and binding to AC elements [49]. The AC elements are cis-acting elements found in most promoters of monolignol biosynthetic genes [36]. In this study, we identified five contigs, two in *Aa *and three in *Am*, which are homologous to R2R3-MYBs regulating wood-related pathways in other plant species. In addition to R2R3-MYBs, NtLIM1 in tobacco had been proven to bind AC elements and its inhibition reduced lignin content [50]. We found one *Am *contig which was highly homologous to tobacco NtLIM1 with 86% identity.

Phylogenetic analysis of the *Acacia *R2R3-MYB proteins with wood-related R2R3-MYBs from *A. thaliana *and other plant species showed that they fall into three groups (Figure 3B). In group one, three *Acacia *R2R3-MYB proteins, namely AauMYB1, AmgMYB1 and AauMYB3 are close homologs of *Arabidopsis *MYB61 and Pine MYB8 while AauMYB1 and AmgMYB1 are orthologs. Pine MYB8 is a close homolog of MYB61 whose overexpression caused ectopic lignin deposition but the exact functions are yet to be known [51,52]. Only one *Am *R2R3-MYB protein (AmgMYB2) belongs to group two which is a close homolog to *Arabidopsis *MYB20 and MYB43. MYB20, MYB42 and MYB43 are activated by NAC master switches to regulate downstream MYB proteins in wood-related pathways [53] whereas MYB85 can induce secondary wall biosynthetic genes [54]. Another member of this group, PineMYB1 is able to bind AC elements [55] and is involved in secondary cell wall deposition [51]. AauMYB2 belongs to group three that clustered together with EgMYB1, AmMYB308, ZmMYB31 and ZmMYB42, indicates an important role in regulating the monolignol biosynthesis pathway. EgMYB1 binds AC element and represses the monolignol biosynthesis pathway [56]. AmMYB308, ZmMYB31 and ZmMYB42 have been shown to affect lignin content by regulating the expression of lignin genes [57,58].

### Identification of microRNA genes and gene targets

For non-model species like *Acacia*, microRNAs (miRNAs) can be identified from the transcriptome data based on homology searches against publicly available databases [59]. We searched for miRNAs by comparing our contigs to known plants miRNA stem-loop sequences downloaded from miRbase [60]. We found nine matching sequences from *Aa *corresponding to eight conserved families (miR319, 396, 162, 160, 168, 166, 172 and 159) and one recently identified family (miR2086). Four of these families (miR319, 396, 2086 and 166) were also found in *Am*. Most predicted miRNA genes such as miR319, miR396, miR162, miR166, miR168, miR172 are highly conserved in plants. The number of miRNAs detected in this study was lower compared to another study [61] because miRNAs are most abundant in leaves and flowers.

**Figure 4:**
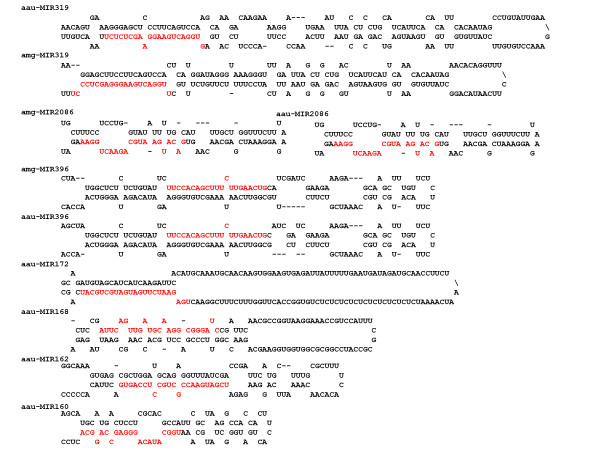
**Stem-loop structures of miRNAs found in *A. auriculiformis *and *A. mangium***.

Blastx results showed that all primary transcripts except miR2086 have no significant hits to any protein-coding gene, suggesting that primary transcript sequences are less conserved in plants. Primary transcripts of miR159 and miR166 were removed from further analysis due to incomplete stem-loop structure and missing mature miRNA sequence. The presence of gap region in the stem loop sequences of miR396, miR160 and miR172 in *Aa *resulted in inaccurate stem-loop structure prediction. Therefore, PCR amplification and sequencing were carried out to fill up the gap. The secondary structures of miR319, miR396, miR2086, miR160, miR162, miR168 and miR172 predicted by Mfold were stable (Figure 4) and all except miR160 have high MFEI values (Table 2). miR2086 is a relatively new family highly expressed in the stem of *M. truncatula *[62]. Blastx indicated that both primary transcripts of miR2086 code for DNA glycosylase (E-value = 0.0). The predicted target of miR2086 is nodulin-like protein suggesting it might play a role in nitrogen fixing pathway. This family is predicted to be a legume-specific miRNA.

**Table 2:** Predicted miRNAs in *A. auriculiformis *and *A. mangium*.

miRNA family	mature miRNA sequence (5'-3')	miRNA mismatch	Length (nt)	MFE	GC %	MFEI
aau-miR319	uuggacugaagggagcucccu	3	197	-68.9	41.1	0.85
aau-miR396	uuccacagcuuucuugaacug	0	146	-63.0	44.5	0.97
aau-miR2086	gacaugaaugcagaacuggaa	0	87	-23.4	39.1	0.69
aau-miR160	uggcauacagggagccaggca	0	88	-29.4	56.8	0.59
aau-miR162	ucgauaaaccucugcauccag	0	103	-40.0	48.5	0.80
aau-miR168	auucaguugaugcaaggcgggauc	2	127	-57.8	59.1	0.77
aau-miR172	ugagaaucuugaugaugcugcau	0	165	-59.6	40.6	0.89
amg-miR319	guggacugaaggaagcucucu	0	182	-82.0	41.2	1.09
amg-miR396	uuccacagcuuucuugaacug	0	145	-63.8	44.1	1.00
amg-miR2086	gacaugaaugcagaacuggaa	0	87	-23.4	39.1	0.69

A total of 512 and 442 contigs in *Aa *and *Am *were predicted to be the targets for 135 and 134 miRNA families found in plants. Blastx results for the predicted targets of several highly conserved miRNAs are indicated in Table 3. We found known targets such as Auxin Response Factor, APETALA 2, F-box protein, Cc-NBS-LRR disease resistance genes and Heat Shock Protein for miRNA 160, 172, and 396. We predicted three wood-related genes, namely flavonol synthase-like, xyloglucan fucosyltransferase and glucan synthase-like genes to be the targets of miR170, miR172 and miR319, respectively, suggesting that miRNAs might be directly involved in the regulation of phenylpropanoid pathway and hemicellulose biosynthesis pathway. Glucan synthase is involved in the synthesis of xyloglucan which make up the β-1,4-glucan backbone while xyloglucan fucosyltransferase adds fructose sidechains to the backbone. Downregulation of flavonol synthase is predicted to redirect the carbon flux towards lignin biosynthesis as flavonoid biosynthesis uses 4-coumaroyl CoA as precursor. Functional analysis of these putative miRNA targets for potential role in wood formation should be studied in the future.

**Table 3:** Predicted miRNA targets in *A. auriculiformis *and *A. mangium*.

miRNA	Known miRNA targets	Blastx ID	Blastx annotation	E-value
160^a^	Auxin Response	XP_002519531.1	Auxin Response factor	0.0
160^b^	Factors	XP_002519531.1	Auxin Response factor	5e-145

170^a^		AAM63621.1	Flavonol synthase-like protein	7e-12

172^a^	APETALA 2	XP_002534399.1	APETALA 2	7e-65
		XP_002527501.1	Signal transducer	1e-88
		XP_002320412.1	F-box protein	2e-146
		XP_002331783.1	Cc-NBS-LRR resistance protein	5e-60
		AAD41092.1	Xyloglucan fucosyltransferase	1e-110
172^b^		XP_002320412.1	F-box protein	0.0
		NP_973532.1	Protein kinase	0.0
		XP_002516311.1	ATP binding protein	4e-158
		NP_001119113.1	Zinc ion binding	0.0
		Q9M5Q1.1	Xyloglucan fucosyltransferase	2e-104

319^a^	TCP transcription factors	NP_187372.4	ATGSL10 (glucan synthase-like 10)	0.0
		AAC16330.1	SAR DNA-binding protein	0.0
319^b^		NP_187372.4	ATGSL10 (glucan synthase-like 10)	0.0

396^a^	Cell proliferation,	AAB99745.1	Heat shock protein 70	0.0
	GRL	XP_002331783.1	Cc-NBS-LRR resistance protein	2e-74
	transcription factors	AAM61431.1	Developmental protein	5e-84
396^b^		NP_195570.1	Metal ion binding protein	7e-64

2086^a^	Unknown	AAC27411.1	Nodulin-like protein	0.0
2086^b^		AAC27411.1	Nodulin-like protein	3e-16

^a ^*A. auriculiformis*

^b ^*A. mangium*

### Detection of Single Nucleotide Polymorphisms (SNPs)

Single Nucleotide Polymorphisms (SNPs) are abundant markers that are suitable for a species with low genetic diversity such as *A. mangium *[63]. For a non-model species without genome sequences, we detected SNPs by mapping all the reads to *de novo *contigs as reference. We used only contigs at least 200 bp long to ensure sufficient flanking region for genotyping purposes. Although paired-end reads provide more accurate alignments, a large fraction of our contigs were too short to effectively utilize the paired end information, so we mapped the reads as single-end data, which substantially increased the number of mappable reads.

By using Bowtie default settings and allowing two mismatches, we detected a total of 30,837 *Aa *and 19,070 *Am *putative SNPs. After applying several filtering parameters to remove low coverage, low confidence, low minor frequency allele and multi-allelic SNPs, the putative SNPs number was further reduced to 16,648 and 9,335, respectively (Table 4). As expected, transition SNPs occur almost twice as frequently as transversion SNPs. One SNP was estimated to occur in every 1,123 bp and 1,704 bp in the *Aa *and *Am *transcriptomes, respectively. Although these SNPs represent only a portion of the *Acacia *transcriptome, this study has provided a better SNPs estimation compared to a previous study [31] which was based on the SNPs variation in two lignin genes. Further investigations are being carried to validate these SNPs which are useful for the construction of *Acacia *hybrid linkage map.

**Table 4:** Summary of SNPs detected in *A. auriculiformis *and *A. mangium*.

	*A. auriculiformis*	*A. mangium*
Number of contigs at least 200 bp	23,850	20,387
Total size of contigs at least 200 bp (bp)	18,701,412	15,903,039
Putative SNPs	30,837	19,070
Filtered SNPs	16,648	9,335
Transition SNPs	10,826	6,064
Transversion SNPs	5,822	3,271
SNP frequency	1 every 1,123 bp	1 every 1,704 bp

## Conclusion

This is the first comprehensive transcriptome-wide analysis of *Acacia auriculiformis *and *Acacia mangium*. Our results provide valuable genetic resources for further investigation of lignin biosynthesis and wood-related pathways in *Acacia *hybrids. As Next Generation Sequencing and analytical techniques improve, whole transcriptome sequencing using short read platforms will be the most cost-effective way for significant discovery of genes, regulatory sequences and markers in previously unstudied plants.

## Methods

### Plant materials and RNA extraction

Plant materials were collected from one *A. auriculiformis *individual (AA6) and one *A. mangium *individual (AM20) growing in the Forest Research Institute Malaysia (FRIM), Kepong. AA6 and AM20 are parents of an *Acacia *hybrid mapping population. Both trees were about 5 years old at the time of sampling. The trees were propagated by marcotting the 4-year-old mother trees in FRIM's field station at Bidor, Perak and planting took place at Bukit Hari field plot in FRIM Kepong in 2004. Three different tissues, namely young stem, intermediate inner bark and old inner bark tissues were sampled. Young stem tissues consisted of ~5 cm of non-lignified stem, starting from the shoot tip. Inner bark tissues from intermediate and old developmental stages were sampled by cutting the largest branch on each tree into two halves. The upper half represented the intermediate stage while the lower half represented the old stage. The halves were further cut into disks about 3 cm each. The outer bark tissues were peeled off and we collected the inner bark tissues by separating it from the sapwood. The inner bark tissues are cut into smaller pieces and immediately frozen in liquid nitrogen and stored at -80°C until further use. RNA extraction was carried out using QIAGEN RNeasy Mini Kit for each tissue. A single RNA sample for each individual was generated from 20 μg RNA samples pooled from each of the three tissues. The quality and quantity of the RNA were evaluated using a Nanodrop ND-100 Spectrophotometer and Agilent Bioanalyzer. The RNA Integrity Number (RIN) value given by Agilent Bioanalyzer was greater than 7.5. RNase inhibitor was added to the RNA samples before sending to Canada's Michael Smith Genome Sciences Center where ribosomal RNA depletion using Invitrogen Ribominus Kit, cDNA synthesis and library construction were carried out. Each sample was subjected to one lane sequencing on an Illumina GAII platform.

### De novo transcriptome assembly and annotation

Raw reads in QSEQ format were filtered and converted to FASTQ format using a AWK command. Ribosomal RNA was removed by mapping to *A. thaliana *25S and 18S ribosomal RNA sequences using MUMMER [64] and filtered by a custom Python script (available upon request). The quality of the filtered reads was assessed using Python script htseq-qa from HTSeq package http://www-huber.embl.de/users/anders/HTSeq/doc/overview.html. The filtered reads were used in *de novo *assembly using SOAPdenovo v1.03 [33] with all default settings except -R option was enabled and the insert size of 180-250 bp was used. SOAPdenovo performed scaffolding using paired-end read information and returned the assemblies in contigs and scaffolds. In this paper, we used the term "contigs" to refer to both contigs and scaffolds. The sequencing depth was estimated based on total length of the reads used in the assembly divided by total size of transcriptome assemblies. The contigs were searched against NCBI Non-redundant Database using Blastn and Blastx (E-value ≤ 1e-10). All the contigs were translated into protein sequences using FrameDP [65]. To compare transcriptomes of *Aa *and *Am*, we mapped single-end filtered reads from both species to both transcriptome assemblies separately using Bowtie-0.12.3 [66] by allowing three mismatches and ignoring quality score. We applied an iterative mapping and filtering approach to the unmappable reads to find out their origins. Using Bowtie and allowing three mismatches, the single-end filtered reads were mapped to the both *Acacia *transcriptomes and other genomes as reference in the following order: its corresponding *de novo *contigs, *de novo *contigs from the other *Acacia *species, *A. thaliana *organelles (TAIR8 mitochondrial and chloroplast genomes) and *M. truncatula *genome (Mt3.0). After each alignment, mapped reads were removed using Bowtie's --un command and mapped to the next reference sequences. The remaining reads were considered as unmapped reads. The raw reads of *Aa *and *Am *were deposited on the NCBI Sequence Read Archive (SRA) with accession number SRR098315 and SRR098314.

### Discovery of monolignol biosynthetic genes and isoforms

All monolignol biosynthetic genes and isoforms were downloaded from the Arabidopsis Monolignol Biosynthesis Gene Families Database [67]. We searched the contigs for homologs of *A. thaliana *genes in monolignol biosynthesis pathway using local NCBI Blast-2.2.23+ blastx algorithm (E-value ≤ 1E-10). The protein sequences of the contigs were double-checked with ExPASy Translate Tool http://expasy.org/tools/dna.html and aligned with the corresponding *A. thaliana *genes using ClustalW2 [68]. NCBI ORF finder [69] was used to search for Open Reading Frame (ORF). The protein sequences were checked for presence of conserved amino acid motifs to distinguish members within the same family. Protein identity shared between the isoforms and the closest *A. thaliana *isoforms were checked using EMBOSS Matcher [70] available at http://mobyle.pasteur.fr/cgi-bin/portal.py?#forms::matcher. The nucleotide sequences were trimmed and deposited at NCBI Transcriptome Shortgun Assembly (TSA) (Additional File 2). Protein and nucleotide sequences of the monolignol genes of *Aa × Am *hybrid, namely PAL, C4H, COMT, CCoAOMT and CAD were downloaded from Genbank [Genbank: AAW78382.1, AAY86361.1, ABD42947.1, ABX75853.1 and ABX75854.1] and aligned to the homologs in *Aa *and *Am *using ClustalW2.

### Identification of wood-related transcription factors

We downloaded 82 transcription factor and transcriptional regulatory families of *A. thaliana *from PlnTFDB database[48]. We searched the translated contigs against this database using local NCBI Blast-2.2.23+ blastp algorithm (E-value ≤ 1E-10). We further analyzed several classes of wood-related transcription factors such as MYB, LIM and HD-ZIPIII. Protein sequences of R2R3-MYB [Genbank: CAE09058.1, CAE09057.1, NP_566467.2, NP_172425.2, NP_567390.4, NP_176797.1, NP_197163.1, ACA33851.1, AAQ62540.1, ABD60280.1, NP_196791.1, NP_567664.1, NP_001106009.1, NP_001105949.1, XP_002313303.1, NP_187463.1, XP_002299944.1 Swiss-Prot: P81395.1, P81393.1], LIM [Genbank: AT1G01780.1, AT1G10200, AT1G39900.1, AT2G45800.1, AT3G61230.1, AT3G55770.1] and HD-ZIPIII [Genbank: AY919616.1-AY919623.1] from other plant species were downloaded from NCBI Protein Database. To generate the phylogenetic tree of R2R3-MYBs family, the full length amino acid sequences of R2R3-MYBs from other plant species and five homologous *Acacia *R2R3-MYBs, namely AauMYB1, AauMYB2, AauMYB3, AmgMYB1, AmgMYB2 [Genbank: JL052980, JL052981, JL052982, JL053003, JL053004, JL053005] were used. The protein sequences of homologous *Acacia *R2R3-MYBs were double-checked with ExPASy Translate Tool http://expasy.org/tools/dna.html. All the sequences were aligned using Bioedit ClustalW and the alignments were manually improved (Additional File 3). The unrooted tree was constructed using MEGA 5 [71] with the neighbour-joining method and 1,000 bootstraps (Poisson model and pairwise deletion).

### Identification of MicroRNA genes and gene targets

Stem-loop sequences of all major plant miRNAs were downloaded from miRbase database. The transcriptomes of *Aa *and *Am *were searched for potential stem-loop miRNAs using local NCBI Blast-2.2.23+ Blastn algorithm (E-value ≤ 1e-10). The matching sequences were trimmed to 1,000 bp before submitting to miRbase search tool to find stem-loop sequences and mature miRNAs. For miR396, miR160 and miR172 in *Aa*, PCR amplification and sequencing were carried out to find the complete stem-loop sequences. Primers flanking the gap region were designed based on primary transcript sequences (Additional file 4). RNA was extracted from inner bark tissues of the *Aa *individual (AA6) using Qiagen RNeasy Plant Mini kit. The quantity and quality of the total RNA was checked using Nanodrop ND-1000 Spectrophotometer and gel electrophoresis. 5 μg of total RNA were treated with DNase and converted to cDNA using Fermentas RevertAid Premium Reverse Transcriptase. The PCR reaction consists of 300 ng cDNA, 1 × PCR buffer, 2 mM MgCl_2_, 0.2 mM dNTP, 0.25 μM of each primer and 1 U Vivantis Taq polymerase. The amplification profile consists of 2 min incubation at 94°C, followed by 35 cycles of 94°C for 30 s, 58°C for 30 s, 72°C for 30 s and a final extension of 72°C for 10 min. The specific PCR products were observed on 1% agarose gel stained with ethidium bromide and purified using Qiagen Gel Extraction kit. The purified PCR products were cloned into Promega pGem-T Easy Vectors and transformed into *E. coli *strain JM109. The transformed bacteria were spread on a LB plate containing amplicilin, IPTG and X-gal before overnight incubation. Five colonies for each plate were selected and grown overnight in LB broth containing amplicilin. PCR amplification using 1 μl of the culture pellet as DNA template were carried out to select three positive colonies for each primer pair. Plasmid was extracted using Qiagen Qiaprep Spin Miniprep kit and sent to First Base Laboratories Sdn. Bhd. (Malaysia) for forward and reverse sequencing using M13 primers. The sequence data were analyzed using Bioedit and gap regions were identified. Stem-loop sequences were extracted to predict secondary structures using Mfold 3.1 http://http://mfold.rna.albany.edu/?q=mfold/RNA-Folding-Form/. The secondary structures were examined visually and compared to the existing structures in the database. We used modified method from Zhang et al. [72] to identify miRNA genes except lower cutoff value of for Minimal Folding Energy Index (MFEI) was set. miRNA genes with complete stem-loop and mature miRNA sequences are available in miRbase database. We assigned prefixes aau- and amg- to represent *A. auriculiformis *and *A. mangium*. The miRNA targets were identified in *Aa *and *Am *transcriptomes by allowing 3 mismatches using a custom search in psRNAtarget http://bioinfo3.noble.org/psRNATarget/.

### Detection of Single-nucleotide Polymorphisms (SNPs)

The filtered reads were mapped back to the reference using Bowtie-0.12.3 by allowing two mismatches. Only contigs at least 200 bp were used as reference. The generated SAM files were exported to Samtools 0.1.7 [73] and converted to BAM format. We called SNPs using Samtools's Pileup command and removed any SNPs with a SNP score less than 20. The putative SNPs were further filtered using the following criteria: 1) Mapping and SNP score more than 100; 2) SNPs must be covered in at least 10 reads; 3) At least three non-reference alleles are present; 4) SNPs must be bi-allelic; 5) Minor allele frequency must be at least 5%; 6) Total frequency of major and minor allele must be at least 0.95. All filtering was done using Awk and Python scripts (available upon request).

## Authors' contributions

MW prepared the samples, performed data analysis and drafted the manuscript. CC assisted in bioinformatics analysis. WR secured funding and coordinated the project. All the authors read and approved the final manuscript.

## Supplementary Material

Additional file 1 **Multiple protein sequence alignments of monolignol biosynthetic genes in *Arabidopsis thaliana*, *A. auriculiformis *and *A. mangium***. The file provides the multiple protein sequence alignments of all ten monolignol biosynthetic genes detected in *A. auriculiformis *and *A. mangium *with corresponding *A. thaliana *genes. Conserved motifs are highlighted in colour. Click here for file

Additional file 2 **Genbank accession numbers of monolignol biosynthetic genes in *A. auriculiformis *and *A. mangium***. The table provides the lengths and accession numbers for the assembled sequences of monolignol biosynthetic genes from *A. auriculiformis *and *A. mangium *that were deposited in NCBI Transcriptome Shortgun Assembly (TSA). Click here for file

Additional file 3 **Multiple protein sequence alignments of R2R3-MYBs in *A. auriculiformis *and *A. mangium *and other species used in phylogenetic tree construction**. R2 and R3 repeats are shown. Click here for file

Additional file 4 **Primer pairs for miRNA stem-loop sequences in *A. auriculiformis***. The table shows the list of primer sequences with product size and annealing temperature used in the amplification of miR160, miR172 and miR396 stem-loop sequencing in *A. auriculiformis*. Click here for file
