# Supplementary material for: Identification of lignin genes and regulatory sequences involved in secondary cell wall formation in Acacia auriculiformis and Acacia mangium via de novo transcriptome sequencing
Source: BMC Genomics. 2011 Jul 5;12:342. doi: 10.1186/1471-2164-12-342 (PMC3161972; doi:10.1186/1471-2164-12-342)
Supplement: Additional file 1 — Multiple protein sequence alignments of monolignol biosynthetic genes in Arabidopsis thaliana, A. auriculiformis and A. mangium. The file provides the multiple protein sequence alignments of all ten monolignol biosynthetic genes detected in A. auriculiformis and A. mangium with corresponding A. thaliana genes. Conserved motifs are highlighted in colour. [file 1471-2164-12-342-S1.DOC]

**Multiple sequence alignments of monolignol biosynthetic genes in *Arabidopsis thaliana*, *Acacia auriculiformis* and *Acacia mangium***

**1) Phenylalanine ammonia lyase (PAL)**

AaPAL1 ------------MEAVANVKATADSFCLSGGVAAADPLNWGVAAESLKGSHLDEVKRMVE 48

AmPAL1 ------------MEAVANVKATADSFCLSGGVAAADPLNWGVAAESLKGSHLDEVKRMVE 48

AmPAL2 ------------------------------------------------------------

AaPAL2 ------------------------------------------------------------

AthPAL1 MEINGAHKSNGGGVDAMLCGGDIKTKNMVIN--AEDPLNWGAAAEQMKGSHLDEVKRMVA 58

AthPAL2 ----------MDQIEAMLCGGGEKTKVAVTTKTLADPLNWGLAADQMKGSHLDEVKKMVE 50

AmPAL3 ---------MEFSKVNGGNNGAFQLCNSGDTVCNGDPLNWGMAAEALKGSHLDQVKRMVE 51

AaPAL3 ------------------------------------------------------------

AthPAL3 --------------------MEFRQPN--ATALS-DPLNWNVAAEALKGSHLEEVKKMVK 37

AthPAL4 --------------------MELCNQNNHITAVSGDPLNWNATAEALKGSHLDEVKRMVK 40

AaPAL1 EFRKPVVRLGGETLTISQVAAIAAHDQGVKVELSESARAGVKASSDWVMDSMNKGTDSYG 108

AmPAL1 EFRKPVVRLGGETLTISQVAAIAAHDQGVKVELSESARAGVKASSDWVMDSMNKGTDSYG 108

AmPAL2 ------------------------------------------------------------

AaPAL2 ------------------------------------------------------------

AthPAL1 EFRKPVVNLGGETLTIGQVAAISTIGNSVKVELSETARAGVNASSDWVMESMNKGTDSYG 118

AthPAL2 EYRRPVVNLGGETLTIGQVAAISTVGGSVKVELAETSRAGVKASSDWVMESMNKGTDSYG 110

AmPAL3 EYRKPVVQIAGTSLTISQVVAIANHDAGVAVELSEEARAGVKASSDWVMESMNKGTDSYG 111

AaPAL3 ------------------------------------------------------------

AthPAL3 DYRKGTVQLGGETLTIGQVAAVASGG--PTVELSEEARGGVKASSDWVMESMNRDTDTYG 95

AthPAL4 EYRKEAVKLGGETLTIGQVAAVARGGGGSTVELAEEARAGVKASSEWVMESMNRGTDSYG 100

AaPAL1 VTTGFGATSHRRTKQGAALQKELIRFLNAGIFGNG---TESCHTLPHSATRAAMLVRINT 165

AmPAL1 VTTGFGATSHRRTKQGAALQKELIRFLNAGIFGNG---TESCHTLPHSATRAAMLVRINT 165

AmPAL2 -----------------------------------------------------MLVRINT 7

AaPAL2 -----------------------------------------------------MLVRINT 7

AthPAL1 VTTGFGATSHRRTKNGVALQKELIRFLNAGIFGST---KETSHTLPHSATRAAMLVRINT 175

AthPAL2 VTTGFGATSHRRTKNGTALQTELIRFLNAGIFGNT---KETCHTLPQSATRAAMLVRVNT 167

AmPAL3 VTTGFGATSHRRTKQGGALQNELIRFLNAGIFGNG---TESSHILPHTATRAAMLVRINT 168

AaPAL3 -----------------------------------------------------MLVRINT 7

AthPAL3 ITTGFGSSSRRRTDQGAALQKELIRYLNAGIFATGNEDDDRSNTLPRPATRAAMLIRVNT 155

AthPAL4 VTTGFGATSHRRTKQGGALQNELIRFLNAGIFGPG--AGDTSHTLPKPTTRAAMLVRVNT 158

**:*:**

AaPAL1 LLQGYSGIRFEILEAITKFLNHNITPCLPLRGTITASGDLVPLSYIAGLLTGRPNSKAVG 225

AmPAL1 LLQGYSGIRFEILEAITKFLNHNITPCLPLRGTITASGDLVPLSYIAGLLTGRPNSKAVG 225

AmPAL2 LLQGYSGIRFEILEALTKFINHNITPCLPLRGTITASGDLVPLSYIAGLLTGRPNSKAVG 67

AaPAL2 LLQGYSGIRFEILEALTKFINHNITPCLPLRGTITASGDLVPLSYIAGLLTGRPNSKAVG 67

AthPAL1 LLQGFSGIRFEILEAITSFLNNNITPSLPLRGTITASGDLVPLSYIAGLLTGRPNSKATG 235

AthPAL2 LLQGYSGIRFEILEAITSLLNHNISPSLPLRGTITASGDLVPLSYIAGLLTGRPNSKATG 227

AmPAL3 LLQGYSGIRFEILEAMTKFLNHNITPCLPLRGTITASGDLVPLSYVAGFLIGRPNSKSIG 228

AaPAL3 LLQGYSGIRFEILEAMTKFLNHNITPCLPLRGTITASGDLVPLSYVAGLLIGRPNSKSIG 67

AthPAL3 LLQGYSGIRFEILEAITTLLNCKITPLLPLRGTITASGDLVPLSYIAGFLIGRPNSRSVG 215

AthPAL4 LLQGYSGIRFEILEAITKLLNHEITPCLPLRGTITASGDLVPLSYIAGLLTGRPNSKAVG 218

****:**********:*.::* :*:* ******************:**:* *****:: *

AaPAL1 TSGELLTPKEAFQLAGIDSDFFELQPKEGLALVNGTAVGSGLASMVLFEANILAVLSEVL 285

AmPAL1 TSGELLTPKEAFQLAGIDSDFFELQPKEGLALVNGTAVGSGLASMVLFEANILAVLSEVL 285

AmPAL2 PNGEALNPKEAFQLAGIESEFFELQPKEGLALVNGTAVGSGLASMVLFEANILAVLAEVI 127

AaPAL2 PNGEALNPKEAFQLAGIESEFFELQPKEGLALVNGTAVGSGLASMALFEANILAVLAEVI 127

AthPAL1 PNGEALTAEEAFKLAGISSGFFDLQPKEGLALVNGTAVGSGMASMVLFETNVLSVLAEIL 295

AthPAL2 PDGESLTAKEAFEKAGISTGFFDLQPKEGLALVNGTAVGSGMASMVLFEANVQAVLAEVL 287

AmPAL3 PDGRVLSPKEAFHLAGINGGFFELQPKEGLALVNGTAVGSALASIVLFETNILGVLSEVM 288

AaPAL3 SDGRVLSPKEAFHLAGINGGFFELQPKEGLALVNGTAVGSALASIVLFETNILGVLSEVM 127

AthPAL3 PSGEILTALEAFKLAGVSS-FFELRPKEGLALVNGTAVGSALASTVLYDANILVVFSEVA 274

AthPAL4 PSGETLTASEAFKLAGVSS-FFELQPKEGLALVNGTAVGSGLASTVLFDANILAVLSEVM 277

..*. *.. ***. **:. **:*:***************.:** .*:::*: *::*:

AaPAL1 SAIFTEVMQGKPEFTDHLTHKLKHHPGQIEAAAIMEHILDGSSYMKAAQKLHEIDPLQKP 345

AmPAL1 SAIFAEVMQGKPEFTDHLTHKLKHHPGQIEAAAIMEHILDGSSYMKAAQKLHEIDPLQKP 345

AmPAL2 SAIFAEVMQGKPEFTDHLTHKLKHHPGQIEAAAIMEHILDGSSYVKAAKKLHEMDPLQKP 187

AaPAL2 SAIFAEVMQGKPEFTDHLTHKLKHHPGQIEAAAIMEHILDGSSYVKAAKKLHEMDPLQKP 187

AthPAL1 SAVFAEVMSGKPEFTDHLTHRLKHHPGQIEAAAIMEHILDGSSYMKLAQKLHEMDPLQKP 355

AthPAL2 SAIFAEVMSGKPEFTDHLTHRLKHHPGQIEAAAIMEHILDGSSYMKLAQKVHEMDPLQKP 347

AmPAL3 SAIFAEVMQGKPEFTDHLTHKLKHHPGQIEAAAIMEHILDGSSYVKAAQNLHEIDPLQKP 348

AaPAL3 SAIFAEVMQGKPEFTDHLTHKLKHHPGQIEAAAIMEHILDGSSYVKAAQNLHEIDPLQKP 187

AthPAL3 SAMFAEVMQGKPEFTDHLTHKLKHHPGQIEAAAIMEHILDGSSYVKEALHLHKIDPLQKP 334

AthPAL4 SAMFAEVMQGKPEFTDHLTHKLKHHPGQIEAAAIMEHILDGSSYVKEAQLLHEMDPLQKP 337

**:*:***.***********:***********************:* * :*::******

AaPAL1 KQDRYALRTSPQWLGPLIEVIRFSTKSIEREINSVNDNPLIDVSRNKALHGGNFQGTPIG 405

AmPAL1 KQDRYALRTSPQWLGPLIEVIRFSTKSIEREINSVNDNPLIDVSRNKALHGGNFQGTPIG 405

AmPAL2 KQDRYALRTSPQWLGPLIEVIRFSTKSIEREINSVNDNPLIDVSRNKALHGGNFQGTPIG 247

AaPAL2 KQDRYALRTSPQWLGPLIEVIRFSTKSIEREINSVNDNPLIDVSRNKALHGGNFQGTPIG 247

AthPAL1 KQDRYALRTSPQWLGPQIEVIRYATKSIEREINSVNDNPLIDVSRNKAIHGGNFQGTPIG 415

AthPAL2 KQDRYALRTSPQWLGPQIEVIRQATKSIEREINSVNDNPLIDVSRNKAIHGGNFQGTPIG 407

AmPAL3 KQDRYALRTSPQWLGPQIEVIRHATKMIEREINSVNDNPLIDVSRGKALHGGNFQGTPIG 408

AaPAL3 KQDRYALRTSPQWLGPQIEVIRHATKMIEREINSVNDNPLIDVSRGKALHGGNFQGTPIG 247

AthPAL3 KQDRYALRTSPQWLGPQIEVIRAATKMIEREINSVNDNPLIDVSRNKAIHGGNFQGTPIG 394

AthPAL4 KQDRYALRTSPQWLGPQIEVIRAATKMIEREINSVNDNPLIDVSRNKALHGGNFQGTPIG 397

**************** ***** :** ******************.**:***********

AaPAL1 VSMDNTRLALASIGKLMFAQFSELVNDFYNNGLPSNLSAGRNPSLDYGFKGAEIAMASYC 465

AmPAL1 VSMDNTRLALASIGKLMFAQFSELVNDFYNNGLPSNLSAGRNPSLDYGFKGAEIAMASYC 465

AmPAL2 VSMDNTRLAIASIGKLMFAQFSELVNDFYNNGLPSNLTASRNPSLDYGFKGAEIAMASYC 307

AaPAL2 VSMDNTRLAIASIGKLMFAQFSELVNDFYNNGLPSNLTASRNPSLDYGFKGAEIAMASYC 307

AthPAL1 VSMDNTRLAIAAIGKLMFAQFSELVNDFYNNGLPSNLTASRNPSLDYGFKGAEIAMASYC 475

AthPAL2 VSMDNTRLAIAAIGKLMFAQFSELVNDFYNNGLPSNLTASSNPSLDYGFKGAEIAMASYC 467

AmPAL3 VSMDNTRLALASIGKLMFAQFSELVNDFYNNGLPSNLSASRYPSLDYGFKGAEIAMASYC 468

AaPAL3 VSMDNTRLALASIGKLMFAQFSELVNDFYNNGLPSNLSASRYPSLDYGFKGAEIAMASYC 307

AthPAL3 VAMDNTRLALASIGKLMFAQFTELVNDFYNNGLPSNLSGGRNPSLDYGLKGAEVAMASYC 454

AthPAL4 VAMDNSRLAIASIGKLMFAQFSELVNDFYNNGLPSNLSGGRNPSLDYGFKGAEIAMASYC 457

*:***:***:*:*********:***************:.. ******:****:******

AaPAL1 SELQYLANPVTSHVQSAEQHNQDVNSLGLISSRKTNEAIEILKLMSSTYLIALCQAIDLR 525

AmPAL1 SELQYLANPVTSHVQSAEQHNQDVNSLGLISSRKTNEAIEILKLMSSTYLIALCQAIDLR 525

AmPAL2 SELQYLANPVTTHVQSAEQHNQDVNSLGLISSRKTNEAIEILKLMSSTFLVALCQAIDLR 367

AaPAL2 SELQYLANPVTTHVQSAEQHNQDVNSLGLISSRKTNEAIEILKLMSSTFLVALCQAIDLR 367

AthPAL1 SELQYLANPVTSHVQSAEQHNQDVNSLGLISSRKTSEAVDILKLMSTTFLVAICQAVDLR 535

AthPAL2 SELQYLANPVTSHVQSAEQHNQDVNSLGLISSRKTSEAVDILKLMSTTFLVGICQAVDLR 527

AmPAL3 SELQFLANPVTNHVQSAEQHNQDVNSLGLISSRKTAEAVEILKIMSSTFLIALCQAIDLR 528

AaPAL3 SELQFLANPVTNHVQSAEQHNQDVNSLGLISSRKTAEAVEILKLMSSTFLIALCQAIDLR 367

AthPAL3 SELQFLANPVTNHVESASQHNQDVNSLGLISSRTTAEAVVILKLMSTTYLVALCQAFDLR 514

AthPAL4 SELQFLANPVTNHVQSAEQHNQDVNSLGLISSRKTAEAVDILKLMSTTYLVALCQAVDLR 517

****:******.**:**.***************.* **: ***:**:*:*:.:***.***

AaPAL1 HLEENLKSTVNSTVSQVAKRTLTTGVNGELHPSRFCERDLLKVVDREHVFTYIDDPTSAT 585

AmPAL1 HLEENLKSTVNSTVSQVAKRTLTTGVNGELHPSRFCERDLLKVVDREHVFAYIDDPTSAT 585

AmPAL2 HLEENLKNTVKNTVSQVAKRTLTTGVNGELHPSRFCEKDLLKVVDREYVFAYADDPCSAL 427

AaPAL2 HLEENLKNTVKNTVSQVAKRTLTTGVNGELHPSRFCEKDLLKVVDREYVFAYADDPCSAL 427

AthPAL1 HLEENLRQTVKNTVSQVAKKVLTTGVNGELHPSRFCEKDLLKVVDREQVYTYADDPCSAT 595

AthPAL2 HLEENLRQTVKNTVSQVAKKVLTTGINGELHPSRFCEKDLLKVVDREQVFTYVDDPCSAT 587

AmPAL3 HLEENLKNTVKNTVNQVAKKVLTMGVNGELLPSRFCEKDLLTAVDREHVFAYIDDPCSAT 588

AaPAL3 HLEENLKNTVKNTVNQVAKKVLTMGVNGELLPSRFCEKDLLTAVDREHVFAYIDDPCSAT 427

AthPAL3 HLEEILKKAVNEVVSHTAKSVLAI------EPFRKHD-DILGVVNREYVFSYVDDPSSLT 567

AthPAL4 HLEENLKKAVKSAVSQVAKRVLTVGANGELHPSRFTERDVLQVVDREYVFSYADDPCSLT 577

**** *:.:*:..*.:.** .*: * * : *:* .*:** *::* *** *

AaPAL1 YPLMQKLRQVLVDHALENGDNEKNSSTSIFQKIAAFEEELKTLLPKEVERARTAYENGNS 645

AmPAL1 YPLMQKLRQVLVDHALENGDNEKNSSTSIFQKIAAFEEELKTLLPKEVERARTAYENGNS 645

AmPAL2 YPLMQKLRQVLVDHALANAENEKNTSTSIFQKIGTFEEELKNVLPKEVESARVAYETGTS 487

AaPAL2 YPLMQKLRQVLVDHALANAENEKNTSTSIFQKIGTFEEELKNVLPKEVESARVAYETGTS 487

AthPAL1 YPLIQKLRQVIVDHALINGESEKNAVTSIFHKIGAFEEELKAVLPKEVEAARAAYDNGTS 655

AthPAL2 YPLMQRLRQVIVDHALSNGETEKNAVTSIFQKIGAFEEELKAVLPKEVEAARAAYGNGTA 647

AmPAL3 YPLRQKLRQVLVDHALQNGDRENDSNTSIFLKIAAFEEELETHLPNEVESARIEVENGKS 648

AaPAL3 YPLRQKLRQVLVDHALQNGDRENDSNTSIFLKIAAFEEELETHLPNEVESARIEVENGKS 487

AthPAL3 NPLMQKLRHVLFDKALAEPEGETD---TVFRKIGAFEAELKFLLPKEVERVRTEYENGTF 624

AthPAL4 YPLMQKLRHILVDHALADPEREANSATSVFHKIGAFEAELKLLLPKEVERVRVEYEEGTS 637

** *:**:::.*:** : : * : ::* **.:** **: **:*** .* *.

AaPAL1 SVPNKIKECRSYPLYKFVREDLGAGLLTG------------------------------- 674

AmPAL1 SVPNKIKECRSYPLYKFVREDLGAGLLTGEKTRSPGE----------------------- 682

AmPAL2 EIPNRIKECRSYPLYKFVREELGTQLLTGERVISPGEECDKVFTALCQGKIIDPLLECVG 547

AaPAL2 EIPNRIKECRSYPLYKFVREELGTQLLTGERVISPGEECDKVFTALCQGKIIDPLLECVG 547

AthPAL1 AIPNRIKECRSYPLYRFVREELGTELLTGEKVTSPGEEFDKVFTAICEGKIIDPMMECLN 715

AthPAL2 PIPNRIKECRSYPLYRFVREELGTKLLTGEKVVSPGEEFDKVFTAMCEGKLIDPLMDCLK 707

AmPAL3 GIPNRIKECRSYPLYRFVREELGTGLLTGEKVRSPGEEFDKVFTAMCEGRLIDPLLECLK 708

AaPAL3 GIPNRIKECRSYPLYRFVREELGTGLLTGEKVRSPGEEFDKVFSAMCEGRLIDPLLECLK 547

AthPAL3 NVANRIKKCRSYPLYRFVRNELETRLLTGEDVRSPGEDFDKVFRAISQGKLIDPLFECLK 684

AthPAL4 AIANRIKECRSYPLYRFVRDELNTELLTGENVRSPGEEFDKVFLAISDGKLIDPLLECLK 697

:.*:**:*******:***::* : ****

AaPAL1 ----------

AmPAL1 ----------

AmPAL2 EWNGAPLPIC 557

AaPAL2 EWNGAPLPIC 557

AthPAL1 EWNGAPIPIC 725

AthPAL2 EWNGAPIPIC 717

AmPAL3 EWNGAPLPIC 718

AaPAL3 EWNGAPLPIC 557

AthPAL3 EWNGAPISIC 694

AthPAL4 EWNGAPVSIC 707

**2) Cinnammate 4-hybroxylase (C4H)**

AaC4H1 ------------------------------FKLPPGPLPVPIFGNWLQVGDDLNHRNLTD 30

AmC4H1 MDFLLLEXXXXXXXXXXXXXXXXXXXXXXXXKLPPGPLPVPIFGNWLQVGDDLNHRNLTD 60

AthC4H MDLLLLEKSLIAVFVAVILATVISKLRGKKLKLPPGPIPIPIFGNWLQVGDDLNHRNLVD 60

AaC4H2 MDLLLLEKTLIGLFVAVVVAIVVSKLRGRRFKLPPGPLPVPIFGNWLQVGDDLNHRNLTD 60

AmC4H2 ---------------AVVVAIVVSKLRGRRFKLPPGPLPVPIFGNWLQVGDDLNHRNLTD 45

******:*:******************.*

AaC4H1 LAKKFGDIFLLRMGQRNLVVVSSPELAKEVLHTQGVEFGSRTRNVVFDIFTGKGQDMVFT 90

AmC4H1 LAKKFGDIFLLRMGQRNLVVVSSPELAKEVLHTQGVEFGSRTRNVVFDIFTGKGQDMVFT 120

AthC4H YAKKFGDLFLLRMGQRNLVVVSSPDLTKEVLLTQGVEFGSRTRNVVFDIFTGKGQDMVFT 120

AaC4H2 LAKKYGDIFLLKMGQRNLVVVSSPELAKEVLHTQGVEFGSRTRNVVFDIFTGKGQDMVFT 120

AmC4H2 LAKKYGDIFLLKMGQRNLXGGFVARVGEGGAAHAGGGVRIQDWNVVFDIFTGKGQDMVFT 105

***:**:***:****** . : : * . : *****************

AaC4H1 VYGEHWRKMRRIMTVPFFTNKVVQQYRQGWENEVDEVVADVKKNPESAKNGVVLRKRLQL 150

AmC4H1 VYGEHWRKMRRIMTVPFFTNKVVQQYRQGWENEVDEVVADVKKNPESAKNGVVLRKRLQL 180

AthC4H VYGEHWRKMRRIMTVPFFTNKVVQQNREGWEFEAASVVEDVKKNPDSATKGIVLRKRLQL 180

AaC4H2 VYGEHWRKMRRIMTVPFFTNKVVQQQREGWENEVASVVEDVKKNPASAAKGIVLRKRLQL 180

AmC4H2 VYGGALEENAADHDGAVFYEQGGSATKGGVGERGGERGGGCEEESRIGDQGDRAEEAAQL 165

*** .: ..* :: . : * . . . :::. . :* .: **

AaC4H1 MMYNNMYRIMFDTRFESEDDPIFQKLRALNGERSRLAQSFDYNYGDFIPILRPFLRGYLK 210

AmC4H1 MMYNNMYRIMFDTRFESEDDPIFQKLRALNGERSRLAQSFDYNYGDFIPILRPFLRGYLK 240

AthC4H MMYNNMFRIMFDRRFESEDDPLFLRLKALNGERSRLAQSFEYNYGDFIPILRPFLRGYLK 240

AaC4H2 MMYNNMYRIMFDRRFESEDDPLFQRLKALNGERSRLAQSFEYNYGDFIPILRPFLRGYLK 240

AmC4H2 MMYNNMYRIMFDRRFESEDDPLFQRLKALNGERSRLAQSFEYNYGDFIPILRPFLRGYLK 225

******:***** ********:* :*:*************:*******************

AaC4H1 ICKEVKETRLKLFKDYFVNERKKLESTKGSTGNNGLKCAIDHILDAQKKGEINEDNVLYI 270

AmC4H1 ICKEVKETRLKLFKDYFVNERKKLESTKGSTENNGLKCAIDHILDAQKKGEINEDNVLYI 300

AthC4H ICQDVKDRRIALFKKYFVDERKQIASSK-PTGSEGLKCAIDHILEAEQKGEINEDNVLYI 299

AaC4H2 ICKEVKETRLKLFKDYFVDERKKLGSTR-SSSNGELKCAIDHILDAQKKGEINEDNVLYI 299

AmC4H2 ICKEVKETRLKLFKDYFVDERKKLGSTR-SSSNGELKCAIDHILDAQKKGEINEDNVLYI 284

**::**: *: ***.***:***:: *:: .: . *********:*::************

AaC4H1 VENINVAAIETTLWSIEWGIAELVNHPEVQKKLRHEMDTVLGVGHLVTEPDTHKLPYLQA 330

AmC4H1 VENINVAAIETTLWSIEWGIAELVNHPEVQKKLRHEMDTVLGXRAPGYRTRHPQAPIPPG 360

AthC4H VENINVAAIETTLWSIEWGIAELVNHPEIQSKLRNELDTVLGPGVQVTEPDLHKLPYLQA 359

AaC4H2 VENINVAAIETTLWSIEWGVAELVNHPEIQKKLRDEIDTVLGPGHQVTEPDTHKLPYLQA 359

AmC4H2 VENINVAAIETTLWSIEWGVAELVNHPEIQKKLRDEIDTVXGTRPPGDGAGHPQTPIPTG 344

*******************:********:*.***.*:*** * . : * .

AaC4H1 VIKETLRLRMAIPLLVPHMNLHDAKLGG-------------------------------- 358

AmC4H1 RDQRDPPSPNGNPIAGPNMNLHDAKLGGYEIPAESKILVNAWWLANNPTQWKNPEEFRPE 420

AthC4H VVKETLRLRMAIPLLVPHMNLHDAKLAGYDIPAESKILVNAWWLANNPNSWKKPEEFRPE 419

AaC4H2 VIKETLRLRMAIPLLVPHMNLNDAKLDGYDIPAESKILVNAWWLANNPAHWKNPEQFRPE 419

AmC4H2 RDQGDPSPANGHPTPGPPQNLNDAKLGGYDIPAESKILVNAWWLANNPTHWKNPEQFRPE 404

: . * * **:**** *

AaC4H1 ------------------------------------------------------------

AmC4H1 RFLEEEAKXXXXXXXXXXXXXXXXXXXXXXXXXXXXXXXXXXGRLVQNFELLPPPGQSKI 480

AthC4H RFFEEESHVEANGNDFRYVPFGVGRRSCPGIILALPILGITIGRMVQNFELLPPPGQSKV 479

AaC4H2 RFLEEESKVEANGNDFRYLPFGVGRRSCPGIILALPILGVTLGRLVQNFELLPPPGQSKI 479

AmC4H2 RFLEEESNVEANGNDFRYLPFGVGRRSCPGIILALPILGVTLGRLVQNFELLPPPGQSKI 464

AaC4H1 --------------------------

AmC4H1 DTAEKGGQFSLHILKHSTIVCKPRSF 506

AthC4H DTSEKGGQFSLHILNHSIIVMKPRNC 505

AaC4H2 DTAEKGGQFSLHILKHSTIVAKPRSF 505

AmC4H2 DTAEKGGQFSLHILKHSTIVAKPPSF 490

**3) 4-coumarate 3-hydroxylase (C3H)**

AmC3H1 MALLLIFISVIGVFLCYQLYQRLRFKLPPGPRPLPVVGNLYDIKPVRFRCFAEWAQSYGP 60

AaC3H1 MALLLIFISVIGVFLCYQLYQRLRFKLPPGPRPLPVVGNLYDIKPVRFRCFAEWAQSYGP 60

AthC3H MSWFLIAVATIAAVVSYKLIQRLRYKFPPGPSPKPIVGNLYDIKPVRFRCYYEWAQSYGP 60

*: :** ::.*...:.*:* ****:*:**** * *:**************: ********

AmC3H1 IISVWFGSTLNVIVSNSELAKEVLKDHDQQLADRHRSRSAAKFSRDGKDLIWADYGPHYV 120

AaC3H1 IISVWFGSTLNVIVSNSELAKEVLKDHDQQLADRHRSRSAAKFSRDGKDLIWADYGPHYV 120

AthC3H IISVWIGSILNVVVSSAELAKEVLKEHDQKLADRHRNRSTEAFSRNGQDLIWADYGPHYV 120

*****:** ***:**.:********:***:******.**: ***:*:************

AmC3H1 KVRKVCTLELFSPKRLEALRPIREDEVTAMVESIYRDSTNPENEGKSVMVKKYLGAVAFN 180

AaC3H1 KVRKVCTLELFSPKRLEALRPIREDEVTAMVESIYRDSTNPENEGKSVMVKKYLGAVAFN 180

AthC3H KVRKVCTLELFTPKRLESLRPIREDEVTAMVESVFRDCNLPENRAKGLQLRKYLGAVAFN 180

***********:*****:***************::**.. ***..*.: ::*********

AmC3H1 NITRLAFGKRFVNSEGIMDEQGVEFKAIVTNGLKLGASLAMAEHIPWLRWMFPLEEGAFA 240

AaC3H1 NITRLAFGKRFVNSEGIMDEQGVEFKAIVTNGLKLGASLAMAEHIPWLRWMFPLEEGAFA 240

AthC3H NITRLAFGKRFMNAEGVVDEQGLEFKAIVSNGLKLGASLSIAEHIPWLRWMFPADEKAFA 240

***********:*:**::****:******:*********::************ :* ***

AmC3H1 KHGARRDRLTRAIMEEHTQARQRSGGAKQHFVDALLTLQDKYDLSEDTIIGLLWDMITAG 300

AaC3H1 KHGARRDRLTRAIMEEHTQARQRSGGAKQHFVDALLTLQDKYDLSEDTIIGLLWDMITAG 300

AthC3H EHGARRDRLTRAIMEEHTLARQKSSGAKQHFVDALLTLKDQYDLSEDTIIGLLWDMITAG 300

:***************** ***:*.*************:*:*******************

AmC3H1 MDTTAISVEWAMAELIKNPRVQQKAQEELDRVIGFERIMIETDFSSLPYLQCVAKEAMRL 360

AaC3H1 MDTTAISVEWAMAELIKNPRVQQKAQEELDRVIGFERIMIETDFSSLPYLQCVAKEAMRL 360

AthC3H MDTTAITAEWAMAEMIKNPRVQQKVQEEFDRVVGLDRILTEADFSRLPYLQCVVKESFRL 360

******:.******:*********.***:***:*::**: *:*** *******.**::**

AmC3H1 HPPTPLMLPHRANANVKIGGYDIPKGSNVHVNVWAVARDPAVWKDPSEFRPERFLEEDVD 420

AaC3H1 HPPTPLMLPHRANANVKIGGYDIPKGSNVHVNVWAVARDPAVWKDPSEFRPERFLEEDXG 420

AthC3H HPPTPLMLPHRSNADVKIGGYDIPKGSNVHVNVWAVARDPAVWKNPFEFRPERFLEEDVD 420

***********:**:*****************************:* *********** .

AmC3H1 MKGHDFRLLPFGAGRRVCPGAQLGINLVTSMLGHLLHHFCWTPAEGIKPEEIDMSENPGL 480

AaC3H1 MKGHDFRLLPFGAGRRVCPGAQLGINLVTSMLGHLLHHFCWTPAEGIKPEEIDMSENPGL 480

AthC3H MKGHDFRLLPFGAGRRVCPGAQLGINLVTSMMSHLLHHFVWTPPQGTKPEEIDMSENPGL 480

*******************************:.****** ***.:* *************

AmC3H1 VTFMKTPLQAVATPRLPSHLYKRVPAEI 508

AaC3H1 VTFMKTPLQAVATPRLPSHLYKRVPAEI 508

AthC3H VTYMRTPVQAVATPRLPSDLYKRVPYDM 508

**:*:**:**********.****** ::

**4) Caffeic acid O-methyltransferase (COMT)**

AmCOMT1 MGSAGETQITPTHVNDEEANLFAMQLASASVLPMILKSALELDLLEIIAKAGPNAQLSPS 60

AaCOMT1 MGSAGETQITPTHVNDEEANLFAMQLASASVLPMILKSALELDLLEIIAKAGPNAQLSPS 60

AthCOMT MGSTAETQLTPVQVTDDEAALFAMQLASASVLPMALKSALELDLLEIMAKNG--SPMSPT 58

***:.***:**.:*.*:** ************** ************:** * : :**:

AmCOMT1 DIASQLPTKNPDAAVMLDRMMRLLACYNVLSSSLRTLPDGKIERLYGLAPVAKYLVKNED 120

AaCOMT1 DIASQLPTKNPDAAVMLDRMMRLLACYNVLSSSLRTLPDGKIERLYGLAPVAKYLVKNED 120

AthCOMT EIASKLPTKNPEAPVMLDRILRLLTSYSVLTCSNRKLSGDGVERIYGLGPVCKYLTKNED 118

:***:******:*.*****::***:.*.**:.* *.*... :**:***.**.***.****

AmCOMT1 GVSIAPLNLMNQDKVLMESWYYLTETVLEGGIPFNKAHGMTSFEYHGKDARFNKVFNKGM 180

AaCOMT1 GVSIAPLNLMNQDKVLMESWYYLTETVLEGGIPFNKAHGMTSFEYHGKDARFNKVFNKGM 180

AthCOMT GVSIAALCLMNQDKVLMESWYHLKDAILDGGIPFNKAYGMSAFEYHGTDPRFNKVFNNGM 178

*****.* *************:*.:::*:********:**::*****.*.*******:**

AmCOMT1 ADHSTITMKKILETYTGFEGLKSLVDVGGGTGAVINTIVSKYPSIKGINFDLPHVIEEAP 240

AaCOMT1 ADHSTITMKKILETYTGFEGLKSLVDVGGGTGAVINTIVSKYPSIKGINFDLPHVIEEAP 240

AthCOMT SNHSTITMKKILETYKGFEGLTSLVDVGGGIGATLKMIVSKYPNLKGINFDLPHVIEDAP 238

::*************.*****.******** **.:: ******.:************:**

AmCOMT1 SFPGVEHVGGDMFVSVPKADAVFMKWICHDWSDEHCVKFLKNCYDALPENGKVIVAECIL 300

AaCOMT1 SYPGVEHVGGDMFVSVPKADAVFMKWICHDWSDEHCVKFLKNCYDALPENGKVIVAECIL 300

AthCOMT SHPGIEHVGGDMFVSVPKGDAIFMKWICHDWSDEHCVKFLKNCYESLPEDGKVILAECIL 298

*.**:*************.**:**********************::***:****:*****

AmCOMT1 PVAPDSSLATKGVVHIDVIMLAHNPGGKERTEKEFEALAKGAGFQGFRVCCSAFNSYIME 360

AaCOMT1 PVAPDSSLATKGVVHIDVIMLAHNPGGKERTEKEFEALAKGAGFQGFRVCCSAFNSYIME 360

AthCOMT PETPDSSLSTKQVVHVDCIMLAHNPGGKERTEKEFEALAKASGFKGIKVVCDAFGVNLIE 358

* :*****:** ***:* **********************.:**:*::* *.**. ::*

AmCOMT1 FLKKP 365

AaCOMT1 FLKKP 365

AthCOMT LLKKL 363

**5) Ferulate 5-hydroxylase (F5H)**

AaF5H1 ----MALLFIVPLFLLLS---LVARYRRRSPFPPGPKGLPIIGNMSMMDQLTHRGLAKLA 53

AmF5H1 -------------------------------------GLPIIGNMSMMDQLTHRGLAKLA 23

AthF5H MLTLMTLIVLVPLLLFLFPHLLLRRQMLLKPYPPGPKGLPVIGNILMMNQFNHRGLAKLS 60

***:***: **:*:.*******:

AaF5H1 SKYGGVFHLKMGFLHMVAISDPDAARQVLQVYDNIFSNRPATIAISYLTYDRADMAFAHY 113

AmF5H1 SKYGGVFHLKMGFXXXXXXXXXXAARQVLQVYDNIFSNRPATIAISYLTYDRADMAFAHY 83

AthF5H RIYGGLLHLRLGFSHIFVVSSPDIARQVLQVQDHVFSNRPTTIAIRYLTYGGSDLAFCNY 120

***::**::** ******* *::*****:**** ****. :*:**.:*

AaF5H1 GPFWRQMRKLCVMKLFSRKRAESWQSVRDEVDTVVSAVANNVGKPVNIGELVFSLTKNII 173

AmF5H1 GPFWRQMRKLCVMKLFSRKRAESWQSVRDEVDTVVSAVANNVGKPVNIGELVFSLTKNII 143

AthF5H GPFWRRMRKLYVMMLFSRKRAESWVSVDEEVHKSVRLVASNVGKPLNICKLAFSLSRDIT 180

*****:**** ** ********** ** :**.. * **.*****:** :*.***:::*

AaF5H1 YRAAFGSSSQEGQ----DEFIGILQEFSKLFGAFNIADFIP-FLGWIDPQGLNSRLAKAR 228

AmF5H1 YRAAFGSSSQEGQ----DEFIGILQEFSKLFGAFNIADFIP-FLGWIDPQXXXXXLAKAR 198

AthF5H FRAAFGSSSSTSDESRLDEFLEIIQEFSKLFGEFNVADYVPSWLSWIDPQGINGRVEKAR 240

:********. .: ***: *:******** **:**::* :*.***** : ***

AaF5H1 ASLDGFIDKIIDEHIHKRTHKSDGFGHEETDMVDELMAFYSVEAKVSDSEDLQNSIQLTK 288

AmF5H1 ASLDGFIDKIIDEHIHKRTHKSDGFGHEETDMVDELMAFYSVEAKVSDSEDLQNSIQLTK 258

AthF5H KSLDGFIESVIDDHLHKKKREHDNV-DEETDMVDQLLAFYEEEVKVNNS-----VTKINL 294

******:.:**:*:**:.:: *.. .*******:*:***. *.**.:* ::.

AaF5H1 DNIKAIIMDVMFGGTETVASAIEWAMTELMRNPEDLKRVQQELANVVGLDRR-VEETDFD 347

AmF5H1 DNIKAIIMDVMFGGTETVASAIEWAMTELMRNPEDLKRVQQELAXXXXXXXX-XXXXXXX 317

AthF5H DNIKGIIMDVMFGGTETVALAIEWVLTEILRSPENMKRVQDELTSVVGLDRWRVEDTHLE 354

****.************** ****.:**::*.**::****:**:

AaF5H1 KLTFLKCALKETLRLHPPIPLLLHETAEDAEVSGYYIPKKSRVMINAWAIGRDRNSWDDP 407

AmF5H1 XXTFLKCALKETLRLHPPIPLLLHETAEDAEVSGYYIPKKSRVM---------------- 361

AthF5H KLTFLKCILKETLRLHPPFPLLLHETVKDTEISGYFIPKGSRVMVNTYALGRDPNSWSDP 414

***** **********:*******.:*:*:***:*** ****

AaF5H1 DTFKXXXXXXXXXXXXXXXXXXXXXXXXXXXXXXXXXXXXXXXXXXXXXXXTFFTVSLGN 467

AmF5H1 ------------------------------------------------------------

AthF5H ESFNPGRFLNPIAPDLKGNNFEFVPFGSGRRSCPGMQLGLYAFELAVAHLLHCFTWSLPD 474

AaF5H1 CPTG---------------------------------- 471

AmF5H1 --------------------------------------

AthF5H GMNPGDVDTVEGPGLTVPKAIPLVAVPTTRLLCPIVVS 512

**6) 4-coumarate:CoA ligase (4CL)**

Aa4CL1 ----MAATETSVNPQT----------------DFIFRSKLPDIYIPKHLPLHSYCFENLA 40

Am4CL1 ----MAATETSVNPQT----------------DFIFRSKLPDIYIPKHLPLHSYCFENLA 40

Ath4CL1 ----MAPQEQAVSQVMEKQSNNNNS-------DVIFRSKLPDIYIPNHLSLHDYIFQNIS 49

Ath4CL2 ----MTTQDVIVNDQNDQKQCSN---------DVIFRSRLPDIYIPNHLPLHDYIFENIS 47

Ath4CL4 --MVLQQQTHFLTKKIDQEDEEEEPSH-----DFIFRSKLPDIFIPNHLPLTDYVFQRFS 53

Aa4CL2 MISVESQQKPDLSDPSPKPASNESNSANSAXXXXXXXSKLPDIPISNHLPLHTYCFENLA 60

Am4CL2 MISVESQQKPDLSDPSPKPASNESNSANSAQPTHIFKSKLPDIPISNHLPLHTYCFENLA 60

Ath4CL3 MITA-ALHEPQIHKPTDTSVVSDDVLPHSPPTPRIFRSKLPDIDIPNHLPLHTYCFEKLS 59

: *:**** *.:**.* * *:.::

Aa4CL1 ----EFGSRPCLINAPTGDIYTYYDVELTARRVASGLNKFGVGQGDVIMVLLPNSPEFVF 96

Am4CL1 ----EFGSRPCLINAPTGDIYTYYDVELTARRVASGLNKFGVGQGDVIMVLLPNSPEFVF 96

Ath4CL1 ----EFATKPCLINGPTGHVYTYSDVHVISRQIAANFHKLGVNQNDVVMLLLPNCPEFVL 105

Ath4CL2 ----EFAAKPCLINGPTGEVYTYADVHVTSRKLAAGLHNLGVKQHDVVMILLPNSPEVVL 103

Ath4CL4 GDGDGDSSTTCIIDGATGRILTYADVQTNMRRIAAGIHRLGIRHGDVVMLLLPNSPEFAL 113

Aa4CL2 ----QYADRPCLIVGSTGKIYSYAEAHLQSRKIAAGFSKLGVKKGDVIMILLQNSAEFAL 116

Am4CL2 ----QFADRPCLIVGSTGKXXXXXXXXXXXXXIAAGFSKLGVKKGDVIMILLQNSAEFAL 116

Ath4CL3 ----SVSDKPCLIVGSTGKSYTYGETHLICRRVASGLYKLGIRKGDVIMILLQNSAEFVF 115

. .*:* ..** :*:.: .:*: : **:*:** *..*..:

Aa4CL1 SFLGASFRGAMTTAANPFFTAAEVAKQAKASKAKLIVTQSNFYEKVKEIDVK-------- 148

Am4CL1 SFLGASFRGAMTTAANPFFTAAEVAKQAKASKAKLIVTQSNFYEKVKDIDVK-------- 148

Ath4CL1 SFLAASFRGATATAANPFFTPAEIAKQAKASNTKLIITEARYVDKIKPLQNDD-----GV 160

Ath4CL2 TFLAASFIGAITTSANPFFTPAEISKQAKASAAKLIVTQSRYVDKIKNLQND------GV 157

Ath4CL4 SFLAVAYLGAVSTTANPFYTQPEIAKQAKASAAKMIITKKCLVDKLTNLKND------GV 167

Aa4CL2 SFFAASMIGAVVTTANPFYTADEIFKQFHAAKAKVIITQSAYVGKLRDHEAGKDIGEDQL 176

Am4CL2 XXXXXXXXXXXXXXXXXXXXXXXXXXXXXXXKAKVIITQSAYVGKLRDHEAGKDIGEDQL 176

Ath4CL3 SFMGASMIGAVSTTANPFYTSQELYKQLKSSGAKLIITHSQYVDKLKN------LGEN-L 168

:*:*:*. *:

Aa4CL1 -LIFVDSP---------PDGHSHFSELSQADEK---DIPEVEINTDDAVALPYSSGTTGL 195

Am4CL1 -LIFVDSP---------PDGHSHFSELSQADEK---DIPEVEINTDDAVALPYSSGTTGL 195

Ath4CL1 VIVCIDDNES----VPIPEGCLRFTELTQSTTEASEVIDSVEISPDDVVALPYSSGTTGL 216

Ath4CL2 LIVTTDSD-------AIPENCLRFSELTQSEEPRVDSIP-EKISPEDVVALPFSSGTTGL 209

Ath4CL4 LIVCLDDDGDNGVVSSSDDGCVSFTELTQADET---ELLKPKISPEDTVAMPYSSGTTGL 224

Aa4CL2 KVITVDDP---------PEKCLHFSVVSEADENE---VPEVEIDPDDPVALPFSSGTTGL 224

Am4CL2 KVITVDDP---------PEKCLHFSVVSEADENE---VPEVEIDPDDPVALPFSSGTTGL 224

Ath4CL3 TLITTDEPT--------PENCLPFSTLITDDETNP-FQETVDIGGDDAAALPFSSGTTGL 219

:: *. : *: : .*. :* .*:*:*******

Aa4CL1 PKGVMLTHKGLVTSIAQQVDGENPNLYFHHEDVILCVLPLFHIYSLNSVLLCGLRAKAAI 255

Am4CL1 PKGVMLTHKGLVTSIAQQVDGENPNLYFHHEDVILCVLPLFHIYSLNSVLLCGLRAKAAI 255

Ath4CL1 PKGVMLTHKGLVTSVAQQVDGENPNLYFHSDDVILCVLPMFHIYALNSIMLCGLRVGAAI 276

Ath4CL2 PKGVMLTHKGLVTSVAQQVDGENPNLYFNRDDVILCVWPMFHIYALNSIMLCSLRIGATI 269

Ath4CL4 PKGVMITHKGLVTSIAQKVDGENPNLNFTANDVILCFLPMFHIYALDALMLSAMRTGAAL 284

Aa4CL2 PKGVILTHKSLATSVAQQVDGENPNLYLKPEDVLLCVLPLFHIFSLNSVLLCALRAGSAV 284

Am4CL2 PKGVILTHKSLATSVAQQVDGENPNLYLKPEDVLLCVLPLFHIFSLNSVLLCALRAGSAV 284

Ath4CL3 PKGVVLTHKSLITSVAQQVDGDNPNLYLKSNDVILCVLPLFHIYSLNSVLLNSLRSGATV 279

****::***.* **:**:***:**** : :**:**. *:***::*::::* .:* :::

Aa4CL1 MLMPKFEINALLGLIQKYKVSIAPVVPPIVLAISKSPDIDKYDLSSVRVLKSGGAPLSKE 315

Am4CL1 MLMPKFEINALLGLIQKYKVSIAPVVPPIVLAISKSPDIDKYDLSSVRVLKSGGAPLSKE 315

Ath4CL1 LIMPKFEINLLLELIQRCKVTVAPMVPPIVLAIAKSSETEKYDLSSIRVVKSGAAPLGKE 336

Ath4CL2 LIMPKFEITLLLEQIQRCKVTVAMVVPPIVLAIAKSPETEKYDLSSVRMVKSGAAPLGKE 329

Ath4CL4 LIVPRFELNLVMELIQRYKVTVVPVAPPVVLAFIKSPETERYDLSSVRIMLSGAATLKKE 344

Aa4CL2 LLMHKFEIGALLELTQKHKVSVAMVVPPLVLALAKNPMVADYDLSSIRLVLSGAAPLGKE 344

Am4CL2 LLMHKFEIGALLELTQKHKVSVAMVVPPLVLALAKNPMVADYDLSSIRLVLSGAAPLGKE 344

Ath4CL3 LLMHKFEIGALLDLIQRHRVTIAALVPPLVIALAKNPTVNSYDLSSVRFVLSGAAPLGKE 339

::: :**: :: *: :*::. :.**:*:*: *.. *****:*.: **.*.* **

Aa4CL1 LEDSVRAKFPKARLGQGYGMTEAGPVLTMSLAFAKEPMEVKAGACGTVVRNAEMKIVDPD 375

Am4CL1 LEDSVRAKFPKARLGQGYGMTEAGPVLTMSLAFAKEPMEVKAGACGTVVRNAEMKIVDPD 375

Ath4CL1 LEDAVNAKFPNAKLGQGYGMTEAGPVLAMSLGFAKEPFPVKSGACGTVVRNAEMKIVDPD 396

Ath4CL2 LEDAISAKFPNAKLGQGYGMTEAGPVLAMSLGFAKEPFPVKSGACGTVVRNAEMKILDPD 389

Ath4CL4 LEDAVRLKFPNAIFGQGYGMTESG-TVAKSLAFAKNPFKTKSGACGTVIRNAEMKVVDTE 403

Aa4CL2 LEDALRSRVPQAVLGQGYGMTEAGPVLSMCLGFAKEAFTTKSGSCGTVVRNAELKVLHPE 404

Am4CL2 LEDALRSRVPQAVLGQGYGMTEAGPVLSMCLGFAKEAXXXXXXXXXTVVRNAELKVLHPE 404

Ath4CL3 LQDSLRRRLPQAILGQGYGMTEAGPVLSMSLGFAKEPIPTKSGSCGTVVRNAELKVVHLE 399

*:*:: :.*:* :********:* .:: .*.***:. **:****:*::. :

Aa4CL1 TSHSLPRGHPGEICIRGDQIMKGYLNDPEATKRTIDEEGWLHTGDIGYIDEDDELFIVDR 435

Am4CL1 TSHSLPRGHSGEICIRGDQIMKGYLNDPEATKRTIDEEGWLHTGDIGFVDEDDELFIVDR 435

Ath4CL1 TGDSLSRNQPGEICIRGHQIMKGYLNNPAATAETIDKDGWLHTGDIGLIDDDDELFIVDR 456

Ath4CL2 TGDSLPRNKPGEICIRGNQIMKGYLNDPLATASTIDKDGWLHTGDVGFIDDDDELFIVDR 449

Ath4CL4 TGISLPRNKSGEICVRGHQLMKGYLNDPEATARTIDKDGWLHTGDIGFVDDDDEIFIVDR 463

Aa4CL2 TGLSLGYNQPGEICIRGHQIMKGYLNNEEATATTIDAEGWLHTGDIGYVDDDDELFIVDR 464

Am4CL2 TGLSLGYNQPGEICIRGHQIMKGYLNNEEATATTIDAEGWLHTGDIGYVDDDDELRPK-- 462

Ath4CL3 TRLSLGYNQPGEICIRGQQIMKEYLNDPEATSATIDEEGWLHTGDIGYVDEDDEIFIVDR 459

* ** .:.****:**.*:** ***: ** *** :*******:* :*:***:

Aa4CL1 LKELIKYKGFQVAPAELEALLLSHPHISDAAVVPMKDEAAGEVPVAFVVRSNGHTQTTED 495

Am4CL1 LKELIKYKGFQVAPAELEALLLSHPHISDAAVVPMKDEAAGEVPVAFVVRSNGHTQTTED 495

Ath4CL1 LKELIKYKGFQVAPAELEALLIGHPDITDVAVVAMKEEAAGEVPVAFVVKSKD-SELSED 515

Ath4CL2 LKELIKYKGFQVAPAELESLLIGHPEINDVAVVAMKEEDAGEVPVAFVVRSKD-SNISED 508

Ath4CL4 LKELIKFKGYQVAPAELEALLISHPSIDDAAVVAMKDEVADEVPVAFVARSQG-SQLTED 522

Aa4CL2 VKELIKFKGFQVPPAELEGLLVSHPSIADAAVVPQKDEAAGEVPVAFVVRSNG-FDLTEE 523

Am4CL2 ------------------------------------------------------------

Ath4CL3 LKEVIKFKGFQVPPAELESLLINHHSIADAAVVPQNDEVAGEVPVAFVVRSNG-NDITEE 518

Aa4CL1 DIKQFVSKQVVFYKRISRVFFIDAIPKXXXXXXXXXXLRAKLAADAAK 543

Am4CL1 DIKQFVSKQVVFYKRISRVFFIDAIPKSPSGKILRKDLRAKLAADAAN 543

Ath4CL1 DVKQFVSKQVVFYKRINKVFFTESIPKAPSGKILRKDLRAKLANGL-- 561

Ath4CL2 EIKQFVSKQVVFYKRINKVFFTDSIPKAPSGKILRKDLRARLANGLMN 556

Ath4CL4 DVKSYVNKQVVHYKRIKMVFFIEVIPKAVSGKILRKDLRAKLETMCSK 570

Aa4CL2 AVKEYIAKRVVFYKRLHRVYFVHAIPKSPSGKILRKD----------- 560

Am4CL2 ------------------------------------------------

Ath4CL3 DVKEYVAKQVVFYKRLHKVFFVASIPKSPSGKILRKDLKAKLC----- 561

**7) Hydroxycinnamoyl-CoA shikimate/quinatehydroxycinnamoyltransferase (HCT)**

AmHCT1 ----------------TPRQALWNSNVDLVVPNFHTPSVYFYRPTG------ADDFFDAE 38

AaHCT1 MIINVKASTMVRPAEETPRQALWNSNVDLVVPNFHTPSVYFYRPTG------ADDFFDAE 54

AthHCT MKINIRDSTMVRPATETPITNLWNSNVDLVIPRFHTPSVYFYRPTG------ASNFFDPQ 54

AmHCT2 --------------------------------------VYFYRPTMASVCAAASSFFDAK 22

AaHCT2 ------------------------------------------------------------

AmHCT1 VMKQALAKALVPFYPMAGRLRRDEDGRVEIDCNGEGVLFVEAETTSLIDDFGDFAPTLEL 98

AaHCT1 VMKQALAKALVPFYPMAGRLRRDEDGRVEIDCNGEGVLFVEAETTSLIDDFGDFAPTLEL 114

AthHCT VMKEALSKALVPFYPMAGRLKRDDDGRIEIDCNGAGVLFVVADTPSVIDDFGDFAPTLNL 114

AmHCT2 VLKEALSKVLVPFYPXXXXXXXXXXXXXXXXXXXXXXXXXXXXXXXXXXXXXXXXPTLEL 82

AaHCT2 ----ALSKVLVSFYPMAGRLRRDXXXXXXXXXXXXXXXXXXXXXXXXXXXFGDFAPTLEL 56

**:*.**.*** ***:*

AmHCT1 RQLIPAVDYSGGIETYPLLVLQVTYFKCGGVSLGVGMQHHAADGFSGLHFINSWSDMARG 158

AaHCT1 RQLIPAVDYSGGIETYPLLXLAGNEFKCGGVSLGVGMQHHAADGFSGLHFINSWSDMARG 174

AthHCT RQLIPEVDHSAGIHSFPLLVLQVTFFKCGGASLGVGMQHHAADGFSGLHFINTWSDMARG 174

AmHCT2 RQLIPAVDYSRGIETYPLLVLQITYFRCGGVSLGVGMQHHVADGCSGLWFINTWSDIARG 142

AaHCT2 RQLIPAVDYSRGIETYPLLVLQITYFRCGGVSLGVGMQHHVADGCSGLWFINTWSDIARG 116

***** **:* **.::*** * . *:***.*********.*** *** ***:***:***

AmHCT1 LDLTLP-PFIDRTLLRARDPPQPVFEHIEYKPPPTMKSPVQT----ASKPGSDNNPP--- 210

AaHCT1 LDLTLP-PFIDRTLLRARDPPQPVFEHIEYKPPPTMKSPVQT----ASKPGSDNNPP--- 226

AthHCT LDLTIP-PFIDRTLLRARDPPQPAFHHVEYQPAPSMKIPLD-----PSKSGPEN------ 222

AmHCT2 LDLTVPLPFIDRTLLRAQDPPRPVFSHIEYKPPPAMITPASSLPPSPAKPGTDSAPPPPP 202

AaHCT2 LDLTVPLPFIDRTLLRAQDPPRPVFSHIEYKPPPAMITPASSLPPSPAKPGTDSAPPPPP 176

****:* **********:***:*.* *:**:*.*:* * . .:*.*.:.

AmHCT1 AAVSIFKMTRDQLNALKAKSKEAGNTITYSSYEMLAGHVWKSTCKARALPDDQETKLYIA 270

AaHCT1 AAVSIFKMTRDQLNALKAKSKEAGNTITYSSYEMLAGHVWKSTCKARALPDDQETKLYIA 286

AthHCT TTVSIFKLTRDQLVALKAKSKEDGNTVSYSSYEMLAGHVWRSVGKARGLPNDQETKLYIA 282

AmHCT2 TAVSIFKMTRDHLNHLKSKSKENGNTINYSSYEMLAGHLWKNVCKAXXXXXXXXXXXXXX 262

AaHCT2 TAVSIFKMTRDHLNHLKSKSKENGNTINYSSYEMLAGHLWKNVCKAXXXXXXXXXXXXXX 236

::*****:***:* **:**** ***:.**********:*:.. **

AmHCT1 TDGRSRLQPPLPPGYFGNVIXXXXXXXXXXXXXQNQLGXAASRIHNALSRMDNEYLRSAL 330

AaHCT1 TDGRSRLQPPLPPGYFGNVIFTATPMAMAGELMSKPTWFAASRIHNALSRMDNEYLRSAL 346

AthHCT TDGRSRLRPQLPPGYFGNVIFTATPLAVAGDLLSKPTWYAAGQIHDFLVRMDDNYLRSAL 342

AmHCT2 XXXXXXXXXXXXXXXXXNVIFTATPVALAGELVSNPTWYAASKIHNSLVRMDNEYLRSAL 322

AaHCT2 XXXXXXXXXXXXXXXXXNVIFTATPVALAGELVSNPTWYAASKIHNSLVRMDNEYLRSAL 295

*** .: **.:**: * ***::******

AmHCT1 DYLELQPDLKALVRGAHTFRCPNLG----------------------------------- 355

AaHCT1 DYLELQPDLKALVRGAHTFRCPNLGITSWVRLPIHDADFGWGRPIFMGPGGIAYEGLSFI 406

AthHCT DYLEMQPDLSALVRGAHTYKCPNLGITSWVRLPIYDADFGWGRPIFMGPGGIPYEGLSFV 402

AmHCT2 DYL--------------------------------------------------------- 325

AaHCT2 DYL--------------------------------------------------------- 298

***

AmHCT1 -------------------------------

AaHCT1 LPHSNNDGSLSVAIAL--------------- 422

AthHCT LPSPTNDGSLSVAIALQSEHMKLFEKFLFEI 433

AmHCT2 -------------------------------

AaHCT2 -------------------------------

**8) Caffeoyl CoA 3-O-methyltransferase (CCoAOMT)**

AthCCoAOMT5 ------------------------------------------------------------

AthCCoAOMT6 ------------------------------------------------------------

AthCCoAOMT2 ------------------------------------------------------------

AmCCoAOMT4 ------------------------------------------------------------

AthCCoAOMT7 ------------------------------------------------------------

AaCCoAOMT1 ---------------------------------------------MASS----------N 5

AmCCoAOMT1 ---------------------------------------------MASS----------N 5

AmCCoAOMT2 ---------------------------------------------MA------------- 2

AaCCoAOMT2 ---------------------------------------------MA------------- 2

AthCCoAOMT1 ---------------------------------------------MATTTTEATKTSSTN 15

AaCCoAOMT3 ---------------------------------------------MASDK---------E 6

AthCCoAOMT3 MTTFSTSFLFLLLVFCLIGSLAADDLQHKSGRDVCSGGSDLRTPDIRLNRPTDSVVGNCP 60

AthCCoAOMT4 ---MSTG---LALNRCSV-SVCRTAVTLLNRPTVSVARS------LKFSR---RLIGNCS 44

AthCCoAOMT5 ----------MDGRLPDKGILKSEA-LKQYIMETTAYPREHELLKELREATIQRYGNLSE 49

AthCCoAOMT6 ----------MANEIPTKGILKSEA-LKQYIMETSAYPREHELLKELRKATVQKYGNLSE 49

AthCCoAOMT2 ----------------------------------------------MVCACMRR----SE 10

AmCCoAOMT4 ----------------------------XYILETSVYPREPEPLKELRHVTANHP--RAL 30

AthCCoAOMT7 -----------MAKDEAKGLLKSEE-LYKYILETSVYPREPEVLRELRNITHNHP--QAG 46

AaCCoAOMT1 GEEKKQSEAGRHQEVGHKSLLQSDA-LYQYILETSVYPREPEPMKELREITAKHP--WNI 62

AmCCoAOMT1 GEEKKQSEAGRHQEVGHKSLLQSDA-LYQYILETSVYPREPEPMKELREITAKHP--WNI 62

AmCCoAOMT2 --DQNQSEAGRHQEVGHKSLLQSDA-LYQYILDTSVYPREPEPMKELREITAKHP--WNI 57

AaCCoAOMT2 --DQNQSEAGRHQEVGHKSLLQSDA-LYQYILDTSVYPREPEPMKELREITAKHP--WNI 57

AthCCoAOMT1 GEDQKQSQNLRHQEVGHKSLLQSDD-LYQYILETSVYPREPESMKELREVTAKHP--WNI 72

AaCCoAOMT3 DGAPKSQASGQHKDLSHKSLLQSDA-LYQYILDTSVYPKEHQCLKELRDLTERHP--WNA 63

AthCCoAOMT3 -TEASPLVMADDEKYGNKMVISLTPRLYDYVLNN---VREHEILKQLREETAIS-----Q 111

AthCCoAOMT4 IAPADPYVVADDDKYGNKQVISLTPRLYDYVLSN---VREPKILRQLREETSKMR--GSQ 99

:

AthCCoAOMT5 MGVPVDESLFLSMLVKIINAKNTIEIGVFTGYSLFTVALALPEDGRITAIDIDQAGYNLG 109

AthCCoAOMT6 MEVPVDEGHFLSMLVKIMNAKNTIEIGVFTGYSLLTTALALPEDGRITAIDIDKEAYEVG 109

AthCCoAOMT2 MEVPVDEGHFLSMLLKIMNAKKTIELGVFTGYSLLTTALALPHDGHVTGIDIDKEAYEMG 70

AmCCoAOMT4 MATAPDAGQIXXXXXXXXXXXXXXXVGVFTGYSLLLTALSIPEDGKITAIDMNRESYEIG 90

AthCCoAOMT7 MATAPDAGQLMGMLLNLVNARKTIEVGVFTGYSLLLTALTLPEDGKVIAIDMNRDSYEIG 106

AaCCoAOMT1 MTTSADEGQFLNMLLKLINAKNTMEIGVYTGYSLLATALALPDDGKILAMDINRENYELG 122

AmCCoAOMT1 MTTSADEGQFLNMLLKLINAKNTMEIGVYTGYSLLATALALPDDGKILAMDINRENYELG 122

AmCCoAOMT2 MTTSADEGQFLNMLLKLINAKNTMEIGVYTGYSLLATALALPEDGKILAMDINKENYELG 117

AaCCoAOMT2 MTTSADEGQFLNMLLKLINAKNTMEIGVYTGYSLLATALALPEDGKILAMDINKENYELG 117

AthCCoAOMT1 MTTSADEGQFLNMLIKLVNAKNTMEIGVYTGYSLLATALALPEDGKILAMDVNRENYELG 132

AaCCoAOMT3 IAVPPDEGQFLNMLVKLINARNTIEIGVFTGYSLLATALALPQDGKILALDLNRADYELG 123

AthCCoAOMT3 IQVSPDQAQLLAMLVEILGAKRCIEVGVYTGYSSLAVALVLPESGRLVACDKDANALEVA 171

AthCCoAOMT4 MQVSPDQAQLLAMLVQMLAAERCIEVGVYTGYSSLAVALVLPESGCLVACERDSNSLEVA 159

: .. * . : :**:**** : .** :*..* : . : : ::.

AthCCoAOMT5 LEFMKKAGVDHKINFIQSDAVRGLDQLLNG---KQEYDFAFVDADKTNYVYFLEKLLKLV 166

AthCCoAOMT6 LEFIKKAGVDHKINFIHSDGLKALDQLVND---KCEFDFAFADADKSSYVNFHERLLKLV 166

AthCCoAOMT2 LEFIKNAGVHHKINFIHSDCLQALDNMLSENP-KPEFDFAFVDADKPNYANMHERLMKLV 129

AmCCoAOMT4 LPIIKKAGVEPXXXXXXXXXXXXXXXXXXXXXXXXXXXFAFIDADKVNYWNYHERLMKLV 150

AthCCoAOMT7 LPVIKKAGVEHKIDFKESEALPALDELLNNKVNEGGFDFAFVDADKLNYWNYHERLIRLI 166

AaCCoAOMT1 RPTLEKAGVAHKVDFREGPALPFLDELVKDEKNHGSFDFIFVDADKDNYLNYHERLLQLV 182

AmCCoAOMT1 RPTLEKAGVAHKVDFREGPALPFLDELVKDEKNHGSFDFIFVDADKDNYLNYHERLLQLV 182

AmCCoAOMT2 LPVIQKAGVAHKITFKEGPALPVLDELVKDEKNHGSYDFIFVDADKDNYLNYHKRLIDLV 177

AaCCoAOMT2 LPVIQKAGVAHKITFKEGPALPVLDELVKDEKNHGSYDFIFVDADKDNYLNYHKRLIDLV 177

AthCCoAOMT1 LPIIEKAGVAHKIDFREGPALPVLDEIVADEKNHGTYDFIFVDADKDNYINYHKRLIDLV 192

AaCCoAOMT3 LPVIEKAGVAHKIDFREGPALPVLDQLLTDEKNKGAFDFIFVDADKENYLNYHKRTIELV 183

AthCCoAOMT3 KRYYELAGVSHKVTVKHGLAAESLMSMIQNGE-ESSYDFAFLDADKAMYQEYFESLLRLV 230

AthCCoAOMT4 KRYYELAGVSHKVNVKQGLAAESLKSMIQNGE-GASYDFAFVDADKRMYQDYFELLLQLV 218

: *** * * **** * : : *:

AthCCoAOMT5 KVGGIIAFDNTLWFGTLIQ-KENEVPGHMRAYREALLEFNKILARDPRVEIAQISIGDGL 225

AthCCoAOMT6 KVGGIIAFDNTLWFGFVAE-DEDGVPEHMREYRAALIEFNKKLALDPRVEVSQISIGDGI 225

AthCCoAOMT2 KVGGVIAFDNTLWSGFVAE-KEENVPVHMRVNRKAFLDLNKRLAADPHVEVSQVSIGDGV 188

AmCCoAOMT4 KVGGIVVYDNT------------------------------------------------- 161

AthCCoAOMT7 KVGGIIVYDNTLWGGSVAEPDSSTP-EWRIEVKKATLELNKKLSADQRVQISQAALGDGI 225

AaCCoAOMT1 KVGG-------------------------------------------------------- 187

AmCCoAOMT1 KVGGVIGYDNTLWNGSVVAPDDAPLRKYVMYYREFVLKLNKALAVDPRIEICMLPVGDGI 242

AmCCoAOMT2 KVGGVIGYDNTLWNGSVVAPPDAPLRKYVRYYRDFVLELNKALAVDPRIEICMLPVGDGV 237

AaCCoAOMT2 KVGGVIGYDNTLWNGSVVAPPDAPLRKYVRYYRDFVLELNKALAVDPRIEICMLPVGDGV 237

AthCCoAOMT1 KIGGVIGYDNTLWNGSVVAPPDAPMRKYVRYYRDFVLELNKALAADPRIEICMLPVGDGI 252

AaCCoAOMT3 KVGGVIAYDNTLWQGSVADPPEAXXXXXXKYFRGFVQELNKALALDSRI----------- 233

AthCCoAOMT3 RVGGVIVIDNVLWHGWVADSTVNDERTISLRN------FNKKLMDDQRVSISMVSIGDGM 284

AthCCoAOMT4 RVGGVIVMDNVLWHGRVSDPMVNDAKTISIRN------FNKKLMDDKRVSISMVPIGDGM 272

::**

AthCCoAOMT5 TLCRRLI 232

AthCCoAOMT6 TLCRRLV 232

AthCCoAOMT2 TLCRRLV 195

AmCCoAOMT4 -------

AthCCoAOMT7 TICRRLY 232

AaCCoAOMT1 -------

AmCCoAOMT1 TLCR--- 246

AmCCoAOMT2 TLCRRIS 244

AaCCoAOMT2 TLCRRIS 244

AthCCoAOMT1 TICRRIS 259

AaCCoAOMT3 -------

AthCCoAOMT3 TICRKR- 290

AthCCoAOMT4 TICRKR- 278

**9) Cinnamayl alcohol dehydrogenase (CAD)**

AthCAD4 -----MGKVLEKEAFGLAAKDESGILSPFSFSRRATGEKDVRFKVLFCGICHTDLSMAKN 55

AthCAD5 -----MGKVLQKEAFGLAAKDNSGVLSPFSFTRRETGEKDVRFKVLFCGICHSDLHMVKN 55

AmCAD2 ----------------------------------------VTFKVLYCGICHSDLHMIRN 20

AthCAD3 MERLSGEKEQSVEAFGWAARDSSGHLSPFVFSRRKTGEEEVRVKVLYCGICHSDLHCLKN 60

AthCAD7 -MVDQ------NKAFGWAANDESGVLSPFHFSRRENGENDVTVKILFCGVCHSDLHTIKN 53

AthCAD8 -MVDQ------NRAFGWAANDESGVLSPFHFSRRENGENDVTVKILFCGVCHSDLHTIKN 53

AthCAD1 -MAKSPETEHPNKVFGWGARDKSGVLSPFHFSRRDNGENDVTVKILFCGVCHTDLHTIKN 59

AaCAD1 ---MGSIEG-ERTTVGWAARDPTGILSPYTFNLRNTGPDDVYIKVHYCGICHSDLHQIKN 56

AmCAD1 ---MGSIEG-ERTTVGWAARDPTGILSPYTFNLRNTGPDDVYIKVYYCGICHSDLHQIKN 56

AthCAD2 ---MGSVEAGEKKALGWAARDPSGVLSPYSYTLRSTGADDVYIKVICCGICHTDIHQIKN 57

AthCAD6 ---MGIMEA-ERKTTGWAARDPSGILSPYTYTLRETGPEDVNIRIICCGICHTDLHQTKN 56

AaCAD3 ------------------------------------------------------------

AthCAD9 ---MSSSESVENECMCWAARDPSGLLSPHTITRRSVTTDDVSLTITHCGVCYADVIWSRN 57

AthCAD4 EWGLTTYPLVPGHEIVGVVTEVGAKVKKFNAGDKVGVGYMAGSCRSCDSCNDGDENYCPK 115

AthCAD5 EWGMSTYPLVPGHEIVGVVTEVGAKVTKFKTGEKVGVGCLVSSCGSCDSCTEGMENYCPK 115

AmCAD2 EWNNSIYPLVPGHEIVGEVIEVGTKVEKFKVGDMVGVGCMVGSCXXXXXXXENLENYCPK 80

AthCAD3 EWHSSIYPLVPGHEIIGEVSEIGNKVSKFNLGDKVGVGCIVDSCRTCESCREDQENYCTK 120

AthCAD7 HWGFSRYPIIPGHEIVGIATKVGKNVTKFKEGDRVGVGVIIGSCQSCESCNQDLENYCPK 113

AthCAD8 HWGFSRYPIIPGHEIVGIATKVGKNVTKFKEGDRVGVGVIIGSCQSCESCNQDLENYCPK 113

AthCAD1 DWGYSYYPVVPGHEIVGIATKVGKNVTKFKEGDRVGVGVISGSCQSCESCDQDLENYCPQ 119

AaCAD1 DLGMSNYPMVPGHEVVGEVIEVGSNVTKFKVGEVVGAGLIVGSCRNCRACKSDIEQYCGK 116

AmCAD1 DLGMSNYPMVPGHEVVGEVIEVGSNVTKFKVGEVVGAGLIVGSCRNCRACKSDIEQYCGK 116

AthCAD2 DLGMSNYPMVPGHEVVGEVLEVGSDVSKFTVGDVVGVGVVVGCCGSCKPCSSELEQYCNK 117

AthCAD6 DLGMSNYPMVPGHEVVGEVVEVGSDVSKFTVGDIVGVGCLVGCCGGCSPCERDLEQYCPK 116

AaCAD3 ------------------------------------------------------------

AthCAD9 QHGDSKYPLVPGHEIAGIVTKVGPNVQRFKVGDHVGVGTYVNSCRECEYCNEGQEVNCAK 117

AthCAD4 MILTSGAKNFDDT-MTHGGYSDHMVCAEDFIIRIPDNLPLDGAAPLLCAGVTVYSPMKYH 174

AthCAD5 SIQTYGFPYYDNT-ITYGGYSDHMVCEEGFVIRIPDNLPLDAAAPLLCAGITVYSPMKYH 174

AmCAD2 MILTYGAVYVDGT-ITYGGYSDLMVADEHFVIRIPDNLPLDAAGPLLCAGITVYSPLRHF 139

AthCAD3 AIATYNGVHHDGT-INYGGYSDHIVVDERYAVKIPHTLPLVSAAPLLCAGISMYSPMKYF 179

AthCAD7 VVFTYNSRSSDGTSRNQGGYSDVIVVDHRFVLSIPDGLPSDSGAPLLCAGITVYSPMKYY 173

AthCAD8 VVFTYNSRSSDGT-RNQGGYSDVIVVDHRFVLSIPDGLPSDSGAPLLCAGITVYSPMKYY 172

AthCAD1 MSFTYNAIGSDGT-KNYGGYSENIVVDQRFVLRFPENLPSDSGAPLLCAGITVYSPMKYY 178

AaCAD1 KIWNYNDVYTDGK-PTQGGFAETMIVDQNFVVKIPEGMSPEQVAPLLCAGVTVYSPLSHF 175

AmCAD1 KIWNYNDVYTDGK-PTQGGFAETMIVDQNFVVKIPEGMSPEQVAPLLCAGVTVYSPLSHF 175

AthCAD2 RIWSYNDVYTDGK-PTQGGFADTMIVNQKFVVKIPEGMAVEQAAPLLCAGVTVYSPLSHF 176

AthCAD6 KIWSYNDVYINGQ-PTQGGFAKATVVHQKFVVKIPEGMAVEQAAPLLCAGVTVYSPLSHF 175

AaCAD3 SVLTFNGVDYDGT-ITKGGYSSNIVVHERYCFLIPKDYPLASAAPLLCAGITVYSPMMRH 59

AthCAD9 GVFTFNGIDHDGS-VTKGGYSSHIVVHERYCYKIPVDYPLESAAPLLCAGITVYAPMMRH 176

. . :. . **::. : . : :* . .******:::*:*: .

AthCAD4 GLD-KPGMHIGVVGLGGLGHVAVKFAKAMGTKVTVISTSERKRDEAVTRLGADAFLVSRD 233

AthCAD5 GLD-KPGMHIGVVGLGGLGHVGVKFAKAMGTKVTVISTSEKKRDEAINRLGADAFLVSRD 233

AmCAD2 GLD-KPGVNVGVXXXXXXXXXXXXXXXXXXXXXXXXXXXPDKEEEAIKNLGADSFVVSRE 198

AthCAD3 GLT-GPDKHVGIVGLGGLGHIGVRFAKAFGTKVTVVSSTTGKSKDALDTLGADGFLVSTD 238

AthCAD7 GMTKESGKRLGVNGLGGLGHIAVKIGKAFGLRVTVISRSSEKEREAIDRLGADSFLVTTD 233

AthCAD8 GMTKESGKRLGVNGLGGLGHIAVKIGKAFGLRVTVISRSSEKEREAIDRLGADSFLVTTD 232

AthCAD1 GMT-EAGKHLGVAGLGGLGHVAVKIGKAFGLKVTVISSSSTKAEEAINHLGADSFLVTTD 237

AaCAD1 GLR-DSGLRGGILGLGGVGHMGVKIAKAMGHHVTVISSSEKKKKEAMEDLGADDYVVSSD 234

AmCAD1 GLR-ESGLRGGILGLGGVGHMGVKIAKAMGHHVTVISSSEKKKKEAMEDLGADDYVVSSD 234

AthCAD2 GLM-ASGLKGGILGLGGVGHMGVKIAKAMGHHVTVISSSDKKKEEAIEHLGADDYVVSSD 235

AthCAD6 GLK-QPGLRGGILGLGGVGHMGVKIAKAMGHHVTVISSSNKKREEALQDLGADDYVIGSD 234

AaCAD3 KMN-QTGKSLGVIGLGGLGHMAVKFGKAFGLNVTIFSTSGSKKEEALTQLGADNFVVSSD 118

AthCAD9 NMN-QPGKSLGVIGLGGLGHMAVKFGKAFGLSVTVFSTSISKKEEALNLLGAENFVISSD 235

: .. *: * :*: ***: ::: :

AthCAD4 PKQMKDAMGTMDGIIDTVSATHPLLPLLGLLKNKGKLVMVGAPAEPLELPVFPLIFGRKM 293

AthCAD5 PKQIKDAMGTMDGIIDTVSATHSLLPLLGLLKHKGKLVMVGAPEKPLELPVMPLIFERKM 293

AmCAD2 RDQMQVRPXXXXXIIDTVSALHPLLPLIASLKSQGKLVMVGVPEKPLELPVFPLLMGRKL 258

AthCAD3 EDQMKAAMGTMDGIIDTVSASHSISPLIGLLKSNGKLVLLGATEKPFDISAFSLILGRKS 298

AthCAD7 SQKMKEAVGTMDFIIDTVSAEHALLPLFSLLKVNGKLVALGLPEKPLDLPIFSLVLGRKM 293

AthCAD8 SQKMKEAVGTMDFIIDTVSAEHALLPLFSLLKVSGKLVALGLLEKPLDLPIFPLVLGRKM 292

AthCAD1 PQKMKAAIGTMDYIIDTISAVHALYPLLGLLKVNGKLIALGLPEKPLELPMFPLVLGRKM 297

AaCAD1 ETQMQKIADSLDYIIDTVPVGHPLEPYLSLLKIDGKLILMGVINTPLQFVSPMVMLGRKT 294

AmCAD1 ETQMQKIADSLDYIIDTVPVGHPLEPYLSLLKIDGKLILMGVINTPLQFVSPMVMLGRKT 294

AthCAD2 PAEMQRLADSLDYIIDTVPVFHPLDPYLACLKLDGKLILMGVINTPLQFVTPLVILGRKV 295

AthCAD6 QAKMSELADSLDYVIDTVPVHHALEPYLSLLKLDGKLILMGVINNPLQFLTPLLMLGRKV 294

AaCAD3 QDQMXALARSFDFIVDTASGDHPFDPYMSLLKTSGVLTLVGFP-SEVKFSPASLNLGMKT 177

AthCAD9 HDQMKALEKSLDFLVDTASGDHAFDPYMSLLKIAGTYVLVGFP-SEIKISPANLNLGMRM 294

:: ::** . *.: * :. ** * :* ..: : : :

AthCAD4 VVGSMVGGIKETQEMVDLAGKHNITADIELISADYVNTAMERLAKADVKYRFVIDVANTM 353

AthCAD5 VMGSMIGGIKETQEMIDMAGKHNITADIELISADYVNTAMERLEKADVRYRFVIDVANTL 353

AmCAD2 VAGTLIGGMKETQEMIDFAAKHNVKPDXXXXXXDYVNKAMERLAKADVKYRFVIDIGNTL 318

AthCAD3 IAGSGIGGMQETQEMIDFAAEHGIKAEIEIISMDYVNTAMDRLAKGDVRYRFVIDISNTL 358

AthCAD7 VGGSQIGGMKETQEMLEFCAKHKIVSDIELIKMSDINSAMDRLAKSDVRYRFVIDVANSL 353

AthCAD8 VGGSQIGGMKETQEMLEFCAKHKIVSDIELIKMSDINSAMDRLVKSDVRYRFVIDVANSL 352

AthCAD1 VGGSDVGGMKETQEMLDFCAKHNITADIELIKMDEINTAMERLAKSDVRYRFVIDVANSL 357

AaCAD1 ITGSFIGSIKETEEMLEFWKEKGLSSMIEMVKMDYINKALERLEKNDVRYRFVVDVAGSK 354

AmCAD1 ITGSFIGSIKETEEMLEFWKEKGLSSMIEMVKMDYINKALERLEKNDVRYRFVVDVAGSK 354

AthCAD2 ISGSFIGSIKETEEVLAFCKEKGLTSTIETVKIDELNIAFERLRKNDVRYRFVVDVAGSN 355

AthCAD6 ITGSFIGSMKETEEMLEFCKEKGLSSIIEVVKMDYVNTAFERLEKNDVRYRFVVDVEGSN 354

AaCAD3 ISGSITGGTKETQEMIDFCAANKIYPNIEMIPIEYINEALQRVVNKDVK----------- 226

AthCAD9 LAGSVTGGTKITQQMLDFCAAHKIYPNIEVIPIQKINEALERVVKKDIKYRFVIDIKNSL 354

: *: *. : *:::: : : : . . :* *::*: : *::

AthCAD4 KPTP------------------- 357

AthCAD5 KPNPNL----------------- 359

AmCAD2 KPDPSRD---------------- 325

AthCAD3 AATRS------------------ 363

AthCAD7 LPESSAEILTEQVDHGVSITSRF 376

AthCAD8 LPESSAEILTEHVDHGVSITSRF 375

AthCAD1 SPP-------------------- 360

AaCAD1 LDDHQ------------------ 359

AmCAD1 LDDDQ------------------ 359

AthCAD2 LVEEAATTTN------------- 365

AthCAD6 LDA-------------------- 357

AaCAD3 -----------------------

AthCAD9 K---------------------- 355

**10) Cinnamoyl CoA reductase (CCR)**

AthCCR1 MPVDVASP-AGKTVCVTGAGGYIASWIVKILLERGYTVKGTVRNPDDPKNTHLRELEGGK 59

AthCCR2 MLVD------GKLVCVTGAGGYIASWIVKLLLERGYTVRGTVRNPTDPKNNHLRELQGAK 54

AaCCR1 MPAAAAXXXXXXTICVTGAGGFIASWIVKLLLERGYTVRGTVRNPDDSKNAHLRELEGAQ 60

AmCCR1 MPAAAASSGSGQTICVTGAGGFIASWIVKLLLERGYTVRGTVRNPDDSKNAHLRELEGAQ 60

* . :*******:*******:********:****** *.** *****:*.:

AthCCR1 ERLILCKADLQDYEALKAAIDGCDGVFHTASPVTDDPEQMVEPAVNGAKFVINAAAEAKV 119

AthCCR2 ERLTLHSADLLDYEALCATIDGCDGVFHTASPMTDDPETMLEPAVNGAKFVIDAAAKAKV 114

AaCCR1 ERLTLHKVDLLDLDSVKSVVNGCDGVIHTASPVTDNPEEMVEPALNGTKNVIIASAEAKV 120

AmCCR1 ERLTLHKVDLLDLDSVKSVVNGCDGVIHTASPVTDNPEEMVEPALNGTKNVIIASAEAKV 120

*** * ..** * ::: :.::*****:*****:**:** *:***:**:* ** *:*:***

AthCCR1 KRVVITSSIGAVYMDPNRDPEAVVDESCWSDLDFCKNTKNWYCYGKMVAEQAAWETAKEK 179

AthCCR2 KRVVFTSSIGAVYMNPNRDTQAIVDENCWSDLDFCKNTKNWYCYGKMLAEQSAWETAKAK 174

AaCCR1 RRVVFTSSIGAVYMDPNRNIDEVVDESCWSNLEYCKNTKNWYCYGKAVAEAAAWDEAKAR 180

AmCCR1 RRVVFTSSIGAVYMDPNRNIDEVVDESCWSNLEYCKNTKNWYCYGKAVAEAAAWDEAKAR 180

:***:*********:***: : :***.***:*::************ :** :**: ** :

AthCCR1 GVDLVVLNPVLVLGPPLQPTINASLYHVLKYLTGSAKTYANLTQAYVDVRDVALAHVLVY 239

AthCCR2 GVDLVVLNPVLVLGPPLQSAINASLVHILKYLTGSAKTYANLTQVYVDVRDVALGHVLVY 234

AaCCR1 GVDLVVVNPVLVLGPLLQSTINASTIHILKYLTGSAKTYANATQAYVHVKDVALAHVLVY 240

AmCCR1 GVDLVVVNPVLVLGPLLQSTINASTIHILKYLTGSAKTYANATQAYVHVKDVALAHVLVY 240

******:******** **.:**** *:************* **.**.*:****.*****

AthCCR1 EAPSASGRYLLAESARHRGEVVEILAKLFPEYPLPTKCKDEKNPRAKPYKFTNQKIKDLG 299

AthCCR2 EAPSASGRYILAETALHRGEVVEILAKFFPEYPLPTKCSDEKNPRAKPYKFTTQKIKDLG 294

AaCCR1 ETPSASGRYLCSERSLHRGELVEVLAKHFPEYPTPTKCSDEKNPRAKPYTFSNKKLKDLG 300

AmCCR1 ETPSASGRYLCSERSLHRGELVEILAKHFPEYPTPTKCSDEKNPRAKPY----------- 289

*:*******: :* : ****:**:*** ***** ****.**********

AthCCR1 LEFTSTKQSLYDTVKSLQEKGHLAPPPPPPSASQESVENGIKIGS 344

AthCCR2 LEFKPIKQSLYESVKSLQEKGHLPLP-------QDSNQNEVIIES 332

AaCCR1 LEFTPVDQCLYDTVKSLQDKGHLPLP-------TKQAEESVQIKS 338

AmCCR1 ---------------------------------------------
