# Supplementary material for: Identification of lignin genes and regulatory sequences involved in secondary cell wall formation in Acacia auriculiformis and Acacia mangium via de novo transcriptome sequencing
Source: BMC Genomics. 2011 Jul 5;12:342. doi: 10.1186/1471-2164-12-342 (PMC3161972; doi:10.1186/1471-2164-12-342)
Supplement: Additional file 2 — Genbank accession numbers of monolignol biosynthetic genes in A. auriculiformis and A. mangium. The table provides the lengths and accession numbers for the assembled sequences of monolignol biosynthetic genes from A. auriculiformis and A. mangium that were deposited in NCBI Transcriptome Shortgun Assembly (TSA). [file 1471-2164-12-342-S2.DOC]

| Genes | Abbreviation | Length of total assembled sequences (bp) | Length of coding region (aa) | TSA accession number* |
| --- | --- | --- | --- | --- |
| Phenylalanine ammonia lyase (PAL) | AaPAL1 | 2,137 | 674 | JL052983 |
|  | AmPAL1 | 2,168 | 682 | JL053007 |
|  | AaPAL2 | 2,260 | 557 | JL052995 |
|  | AmPAL2 | 2,097 | 557 | JL053008 |
|  | AaPAL3 | 2,289 | 557 | JL052993, JL052994 |
|  | AmPAL3 | 2,306 | 718 | JL053006 |
| Cinnammate 4-hybroxylase (C4H) | AaC4H1 | 1,076 | 358 | JL052987 |
|  | AmC4H1 | 1,560 | 506 | JL053017 |
|  | AaC4H2 | 1,735 | 505 | JL052984 |
|  | AmC4H2 | 1,653 | 490 | JL053010 |
| 4-coumarate 3-hydroxylase (C3H) | AaC3H1 | 1,725 | 508 | JL052991 |
|  | AmC3H1 | 1,783 | 508 | JL053015 |
| Caffeic acid O-methyltransferase (COMT) | AaCOMT1 | 1,218 | 365 | JL052986 |
|  | AmCOMT1 | 1,300 | 365 | JL053012 |
| Ferulate 5-hydroxylase (F5H) | AaF5H1 | 1,544 | 471 | JL053001 |
|  | AmF5H1 | 1,085 | 361 | JL053020 |
| 4-coumarate: CoA ligase (4CL) | Aa4CL1 | 1,753 | 543 | JL052992 |
|  | Am4CL1 | 1,753 | 543 | JL053009 |
|  | Aa4CL2 | 1,740 | 560 | JL053002 |
|  | Am4CL2 | 1,443 | 462 | JL053021, JL053022, JL053023 |
| Hydroxycinnamoyl-CoA shikimate/  quinatehydroxy-cinnamoyl-transferase (HCT) | AaHCT1 | 1,447 | 422 | JL053000 |
|  | AmHCT1 | 1,081 | 355 | JL053019 |
|  | AaHCT2 | 1,465 | 298 | JL052998, JL052999 |
|  | AmHCT2 | 1,011 | 325 | JL053018 |
| Caffeoyl CoA 3-O-methyltransferase (CCoAOMT) | AaCCoAOMT1 | 615 | 187 | JL052989 |
|  | AmCCoAOMT1 | 796 | 246 | JL053016 |
|  | AaCCoAOMT2 | 746 | 244 | JL052988 |
|  | AmCCoAOMT2 | 894 | 244 | JL053013 |
|  | AaCCoAOMT3 | 1,213 | 233 | JL052996 |
|  | AmCCoAOMT4 | 503 | 161 | JL053027 |
| Cinnamayl alcohol dehydrogenase (CAD) | AaCAD1 | 1,302 | 359 | JL052985 |
|  | AmCAD1 | 1,321 | 359 | JL053011 |
|  | AmCAD2 | 1,390 | 325 | JL053024, JL053025, JL053026 |
|  | AaCAD3 | 940 | 226 | JL052997 |
| Cinnamoyl CoA reductase (CCR) | AaCCR1 | 1,323 | 338 | JL052990 |
|  | AmCCR1 | 882 | 289 | JL053014 |

Genbank accession numbers of *Acacia auriculiformis* and *Acacia mangium* monolignol biosynthetic genes

*If the assemblies contain more than 10% Ns or 14Ns in a row, they are split into contigs not shorter than 200bp and gaps were trimmed before submission to NCBI Transcriptome Shortgun Assembly (TSA).
