# Supplementary material for: Identification of lignin genes and regulatory sequences involved in secondary cell wall formation in Acacia auriculiformis and Acacia mangium via de novo transcriptome sequencing
Source: BMC Genomics. 2011 Jul 5;12:342. doi: 10.1186/1471-2164-12-342 (PMC3161972; doi:10.1186/1471-2164-12-342)
Supplement: Additional file 3 — Multiple protein sequence alignments of R2R3-MYBs in A. auriculiformis and A. mangium and other species used in phylogenetic tree construction. R2 and R3 repeats are shown. [file 1471-2164-12-342-S3.DOC]

Multiple sequence alignments of R2R3-MYBs in *Acacia auriculiformis* and *Acacia mangium* with other species

**R2 Repeat**

AthMYB61 MGRHSCCY-- ---------- ----KQKLRK GLWSPEEDEK LLTHITNHGH

PtMYB8 MGRHSCCY-- ---------- ----KQKLRK GLWSPEEDEK LVRHITKYGH

AauMYB3 MGRHSCCY-- ---------- ----KQKLRK GLWSPEEDEK LLRHITKYGH

AmgMYB1 XXXXXXXX-- ---------- ----XXXXXK GLWSPEEDEK LLTHVTKYGH

AauMYB1 MGRHSCCY-- ---------- ----KQKLRK GLWSPEEDEK LLTHVTKYGH

EgMYB2 MGMAMGIKEK A--SSNP--- ---HNHKLRK GLWSPEEDEK LMRYMLTNGQ

PtMYB4 MSCTTGGLSS PVS------- ----KPKLRK GLWSPEEDDK LINYMMKNGQ

AthMYB46 MRKPEVAI-- --AASTH--- ---QVKKMKK GLWSPEEDSK LMQYMLSNGQ

PtrMYB3 MRKPDLMA-R DRVPINNNM- ---NRAKLRK GLWSPEEDEK LIQYMLTNGQ

PtrMYB20 MRKPDLVG-K DRVPSNSSI- ---NKAKLRK GLWSPEEDEK LIKYMLTNGQ

AthMYB83 MRKPDITTIR DKGKPNHACG GNNNKPKLRK GLWSPDEDEK LIRYMLTNGQ

AthMYB42 MGRQPCCD-- ---------- ----KLMVKK GPWTAEEDKK LINFILTNGH

AthMYB20 MGRQPCCD-- ---------- ----KVGLKK GPWTAEEDRK LINFILTNGQ

AthMYB43 MGRQPCCD-- ---------- ----KVGLKK GPWTIEEDKK LINFILTNGH

AmgMYB2 XXXXXXXX-- ---------- ----XXXLKK GPWTADEDKK LINFILTNGQ

AthMYB85 MGRQPCCD-- ---------- ----KLGVKK GPWTVEEDKK LINFILTNGH

PtMYB1 MGRQPCCD-- ---------- ----KVGLKK GPWTAEEDRK LVNFITLHGH

AthMYB26 MGHHSCCN-- ---------- ----KQKVKR GLWSPEEDEK LINYINSYGH

AauMYB2 MGRSPCCE-- ---------- ----KAHTNK GAWTKEEXXX XXXXXXXXXX

EgMYB1 MGRSPCCE-- ---------- ----KAHTNK GAWTKEEDDK LIAYIRAHGE

AmMYB308 MGRSPCCE-- ---------- ----KAHTNK GAWTKEEDDR LVAYIRAHGE

ZmMYB42 MGRSPCCE-- ---------- ----KAHTNR GAWTKEEDER LVAYVRAHGE

ZmMYB31 MGRSPCCE-- ---------- ----KAHTNK GAWTKEEDER LVAHIRAHGE

AmMYB330 MGRSPCCE-- ---------- ----KAHTNK GAWTKEEDQR LINYIRAHGE

AthMYB61 GCWSSVPKLA G--------- LQRCGKSCRL RWINYLRPDL KRGAFSPEEE

PtMYB8 GCWSAVPKQA G--------- LQRCGKSCRL RWINYLRPDL KRGTFSPQEE

AauMYB3 GCWSSVPKQA G--------- LQRCGKSCRL RWINYLRPDL KRGTFSQEEE

AmgMYB1 GCWSSVPKQA G--------- LQRCGKSCRL RWINYLRPDL KRGTFSQEEE

AauMYB1 GCWSSVPKQA G--------- LQRCGKSCRL RWINYLRPDL KRGAFSQEEE

EgMYB2 GCWSDIARNA G--------- LQRCGKSCRL RWINYLRPDL KRGAFSPQEE

PtMYB4 GCWSDVAKQA G--------- LQRCGKSCRL RWINYLRPDL KRGAFSPQEE

AthMYB46 GCWSDVAKNA G--------- LQRCGKSCRL RWINYLRPDL KRGAFSPQEE

PtrMYB3 GCWSEIARNA G--------- LQRCGKSCRL RWINYLRPDL KRGAFSPQEE

PtrMYB20 GCWSDIARNA G--------- LQRCGKSCRL RWINYLRPDL KRGAFSPQEE

AthMYB83 GCWSDIARNA G--------- LLRCGKSCRL RWINYLRPDL KRGSFSPQEE

AthMYB42 CCWRALPKLA GLRRCGKSCR LRWT------ ---NYLRPDL KRGLLSDAEE

AthMYB20 CCWRAVPKLS GLLRCGKSCR LRWT------ ---NYLRPDL KRGLLSDYEE

AthMYB43 CCWRALPKLS GLLRCGKSCR LRWI------ ---NYLRPDL KRGLLSEYEE

AmgMYB2 CCWRTVPKLA GLLRCGKSCR LRWT------ ---NYLRPDL KRGLLSEYEE

AthMYB85 CCWRALPKLA GLRRCGKSCR LRWT------ ---NYLRPDL KRGLLSHDEE

PtMYB1 GCWREVPKLA XXXXXXXXXX XXXX------ ---XXXXXXX XXXXXXXSEE

AthMYB26 GCWSSVPKHA GTYTHIHGFC LQRCGKSCRL RWINYLRPDL KRGSFSPQEA

AauMYB2 XXXXXXXXAA G--------- LLRCGKSCRL RWINYLRPDL KRGNFTEEED

EgMYB1 GCWRSLPKAA G--------- LLRCGKSCRL RWINYLRPDL KRGNFTEEED

AmMYB308 GCWRSLPKAA G--------- LLRCGKSCRL RWINYLRPDL KRGNFTEEED

ZmMYB42 GCWRSLPRAA G--------- LLRCGKSCRL RWINYLRPDL KRGNFTADED

ZmMYB31 GCWRSLPKAA G--------- LLRCGKSCRL RWINYLRPDL KRGNFTEEED

AmMYB330 GCWRSLPKAA G--------- LLRCGKSCRL RWINYLRPDL KRGNFTEEED

**R3 Repeat**

AthMYB61 NLIVELHAVL GNRWSQIASR LPGRTDNEIK NLWNSSIKKK LKQRGIDPNT

PtMYB8 NLIVELHSVL GNRWSQIATH LPGRTDNEIK NLWNSCIKKK LRQRGIDPNT

AauMYB3 NLIIELHAVL GNRWSQIAAQ LPGRTDNEIK NLWNSCLKKK LRQRGIDPVT

AmgMYB1 NLIIELHAVL GNRWSQIAAQ LPGRTDNEIK NLWNSCLKKK LKQRGIDPVT

AauMYB1 NLIIELHAVL GNRWSQIAAQ LPGRTDNEIK NLWNSCLKKK LKQRGIDPVT

EgMYB2 ELIVHLHNIL GNRWSQIAAR LPGRTDNEIK NFWNSTLKKR LKMN-----S

PtMYB4 HWIIHLHSIL GNRWSQIAAR LPGRTDNEIK NFWNSCIKKK LKHLS---AS

AthMYB46 DLIIRFHSIL GNRWSQIAAR LPGRTDNEIK NFWNSTIKKR LKKMS----D

PtrMYB3 ELIIHLHSIL GNRWSQIAAR LPGRTDNEIK NFWNSTLKKR FKINS----T

PtrMYB20 ELIIHLHTIL GNRWSQIAAR LPGRTDNEIK NFWNSTLKKR LKINN----T

AthMYB83 DLIFHLHSIL GNRWSQIATR LPGRTDNEIK NFWNSTLKKR LKNNSNNNTS

AthMYB42 QLVIDLHALL GNRWSKIAAR LPGRTDNEIK NHWNTHIKKK LLKMEIDPST

AthMYB20 KMVIDLHSQL GNRWSKIASH LPGRTDNEIK NHWNTHIKKK LRKMGIDPLT

AthMYB43 QKVINLHAQL GNRWSKIASH LPGRTDNEIK NHWNTHIKKK LRKMGIDPLT

AmgMYB2 KMVIDLHAQL GNRWSKIASH LPGRTDNEIK NHWNTHIKKK LKKMGIDPVT

AthMYB85 QLVIDLHANL GNKWSKIASR LPGRTDNEIK NHWNTHIKKK LLKMGIDPMT

PtMYB1 KLIIDLHAAI GNRWSRIAAQ LPGRTDNEIK NYWNTRIKKK LRQMGIDPVT

AthMYB26 ALIIELHSIL GNRWAQIAKH LPGRTDNEVK NFWNSSIKKK LMSHHHHGHH

AauMYB2 ELIIKLHSLL GNKWSLIAGR LPGRTDNEIK NYWNTHXXXX XXXXXXDPST

EgMYB1 EIIIKLHSLL GNKWSLIAGR LPGRTDNEIK NYWNTHIRRK LLNRGIDPAT

AmMYB308 ELIIKLHSLL GNKWSLIAGR LPGRTDNEIK NYWNTHIRRK LLSRGIDPTT

ZmMYB42 DLIVKLHSLL GNKWSLIAAR LPGRTDNEIK NYWNTHIRRK LLGSGIDPVT

ZmMYB31 ELIVKLHSVL GNKWSLIAGR LPGRTDNEIK NYWNTHIRRK LLSRGIDPVT

AmMYB330 EIIIKLHSLL GNKWSLIAGA LPGRTDNEIK NYWNTHIKRK LVSRGIDPQT

AthMYB61 HKPISEVESF -------SDK ---------- ---------- ----------

PtMYB8 HRPLSEVNAE -------AGD ---------- ---------- ----------

AauMYB3 HKPLLEVENN -------GDN QDRDGEKVGG VSSNELNLLK SESSRSDGAS

AmgMYB1 HKPLSEVQN- -------GDD KDKE------ --LNELKLLK SESPSSDATK

AauMYB1 HKPLSEVQN- -------GDD KDKE------ --LNELKLLK SESPSSDATK

EgMYB2 ATSSSNES-- -------DLS ---------- ---------- ----------

PtMYB4 TNNSKSISAP -------NRT ---------- ---------- ----------

AthMYB46 TSNLINNS-- --------SS ---------- ---------- ----------

PtrMYB3 STSSPNDSS- -------DSS ---------- ---------- ----------

PtrMYB20 STSSPNDS-- -------DSS ---------- ---------- ----------

AthMYB83 SGSSPNNSNS -------NSL ---------- ---------- ----------

AthMYB42 HQPLN----- ---------- ---------- ---------- ----------

AthMYB20 HKPLS----- ---------- ---------- ---------- ----------

AthMYB43 HKPLSEQEAS -------QQ- ---------- ---------- ----------

AmgMYB2 HKPLS----- ---------- ---------- ---------- ----------

AthMYB85 HQPLN----- ---------- ---------- ---------- ----------

PtMYB1 HKPLTQMQMQ -------ST- ---------- ---------- ----------

AthMYB26 HHHLSSMASL -------LTN ---------- ---------- ----------

AauMYB2 HRPLNDSTNS AQNNQEAATT ---------- ---------- ----------

EgMYB1 HRLINEPAQD ----HHDEPT ---------- ---------- ----------

AmMYB308 HRSINDGTAS ----QDQVTT ---------- ---------- ----------

ZmMYB42 HRRVAGGAAT -------TIS ---------- ---------- ----------

ZmMYB31 HRPVTEHHAS -------NIT ---------- ---------- ----------

AmMYB330 HRSLNSATTT -------ATA ---------- ---------- ----------

AthMYB61 ----DKPTTS -----NNK-- ---------- RSGNDHK--- ----------

PtMYB8 ----SKNDNS -----NKKVE TQAAMDESHV SAGNEFKHLN AIPRADTANP

AauMYB3 SYEHNRPCSS TIIASYNK-- ---------- AYAHDME--- ----------

AmgMYB1 AYTPIMEGCS SLKYSNX--- ---------- ---------- ----------

AauMYB1 AYTPIMEGCS SLXXXXX--- ---------- ---------- ----------

EgMYB2 ----NPQDIA -----AG--- ---------- ---------- ----------

PtMYB4 ----STMNSS -----ITP-- ---------- ---------- ----------

AthMYB46 ----SPNTAS -----DSS-- ---------- ---------- ----------

PtrMYB3 ----EPRDHV -----VGN-- ---------- ---------- ----------

PtrMYB20 ----EPRDHA -----IGN-- ---------- ---------- ----------

AthMYB83 ----DPRDQH -----VDM-- ---------- ---------- ----------

AthMYB42 ---------- ---------- ---------- ---------- ----------

AthMYB20 ---------- ---------- ---------- ---------- ----------

AthMYB43 ---------- -----AQG-- ---------- ---------- ----------

AmgMYB2 ---------- ---------- ---------- ---------- ----------

AthMYB85 ---------- ---------- ---------- ---------- ----------

PtMYB1 ---------- -----PAQ-- ---------- ---------- ----------

AthMYB26 ----LPYHNG -----FNP-- ---------- ---------- ----------

AauMYB2 ----ISFAS- ---------- ---------- ---------- ----------

EgMYB1 ----ISFAA- ---------- ---------- ---------- ----------

AmMYB308 ----ISFSN- ---------- ---------- ---------- ----------

ZmMYB42 ----FQPSP- ---------- ---------- ---------- ----------

ZmMYB31 ----ISFET- ---------- ---------- ---------- ----------

AmMYB330 ----TPTVN- ---------- ---------- ---------- ----------

AthMYB61 ---------- ---------- ---------- ------SPSS SSATN-----

PtMYB8 KFFHVPVEDN TLIASDSQAM LQNGFINSNS TTTTTTATST ASAANFSLP-

AauMYB3 ---------- ---------- ---------- ------GSSS TSKINTPTNF

AmgMYB1 ---------- ---------- ---------- -------XXX XXXXXXXXXX

AauMYB1 ---------- ---------- ---------- ---------- ---NTNLPTH

EgMYB2 ---------- ---------- ---------- ---------- ----------

PtMYB4 ---------- ---------- ---------- ---------- ----------

AthMYB46 ---------- ---------- ---------- ---------- ----------

PtrMYB3 ---------- ---------- ---------- ---------- ----------

PtrMYB20 ---------- ---------- ---------- ---------- ----------

AthMYB83 ---------- ---------- ---------- -------GGN ---ST-----

AthMYB42 ---------- ---------- ---------- ---------- ---KV-----

AthMYB20 ---------- ---------- ---------- ---------- -IVEK-----

AthMYB43 ---------- ---------- ---------- -------RKK SLVPH-----

AmgMYB2 ---------- ---------- ---------- ---------- ----------

AthMYB85 ---------- ---------- ---------- ---------- ---QE-----

PtMYB1 ---------- ---------- ---------- -------TLL LQEND-----

AthMYB26 ---------- ---------- ---------- -------TTV DDESS-----

AauMYB2 ---------- ---------- ---------- ---------- ----------

EgMYB1 ---------- ---------- ---------- ---------- ----------

AmMYB308 ---------- ---------- ---------- ---------- ----------

ZmMYB42 ---------- ---------- ---------- ---------- ----------

ZmMYB31 ---------- ---------- ---------- ---------- ----------

AmMYB330 ---------- ---------- ---------- ---------- ----------

AthMYB61 -------QDF FLER------ -PSDLSDYFG -FQKLN---- -FNSNLGLSV

PtMYB8 -------KEF FLERFNSVNA TPTSVEAGF- NFINQTTSTQ GFTGERDQKL

AauMYB3 ISSDCSTKDL FLDT------ -YTTTSDFMT NFPLHN---- -INYNNNNPP

AmgMYB1 XXXXXXXXDG FTTS------ -YTT-SGLMC NFPLQ----- -MNYAS----

AauMYB1 CCSKDLLPHG FTTS------ -YTT-SGLMC NFPLQ----- -MNYAS----

EgMYB2 ---------- ---------- ---IMPSFHA -QYDVL---- -AT----CMD

PtMYB4 ---------- ---------- ---FSESSAE -PLEVM---- -AT-------

AthMYB46 ---------- ---------- ---------- ---------- --------SN

PtrMYB3 ---------- ---------- ---IMP--MH -DHDVM---- -TL----CKD

PtrMYB20 ---------- ---------- ---IMP--TH -DPDTM---- -TL----CMD

AthMYB83 ---------- ---------- --SLMDDYHH -DENMM---- -TVGNTMRMD

AthMYB42 ---------- ---------- --FTDTN--- --LVD----- ---KSETSSK

AthMYB20 ---------- ---------- --EDEEPLKK -LQNN----- ---TVPFQET

AthMYB43 ---------- ---------- --DDKNPKQD -QQTK----- ---DEQEQHQ

AmgMYB2 ---------- ---------- ---DQQQTQN -NPAS----- ---RINQVQT

AthMYB85 ---------- ---------- --PSNIDNSK -TIPS----- ---NPDDVSV

PtMYB1 ---------- ---------- --TEQKQQEQ -HNEP----- ---DPDQNQS

AthMYB26 ---------- ---------- --RFMSNIIT -NTNPN---- -FITPSHLSL

AauMYB2 ---------- ---------- ---------- -HVKQE---- -TDN------

EgMYB1 ---------- ---------- ---------- -NSKEI---- -KEM------

AmMYB308 ---------- ---------- ---------- -ANSKE---- -ED-------

ZmMYB42 ---------- ---------- ---------- -NSAAA---- -AA-------

ZmMYB31 ---------- ---------- ---------- -EVAAA---- -AR-------

AmMYB330 ---------- ---------- ---------- -NSCLD---- -FRT------

AthMYB61 TTDSSLCSMI PPQFSPGNMV GSVLQTP--- ---------- ----------

PtMYB8 IDNPVLWVLQ APNRSVGFPT ENLMPWPGQG LAKAVSDAFS DFSSDVCDYN

AauMYB3 TNDSCRWFST QTQARPFDIN TTEFTFP--- ---------- ----------

AmgMYB1 TSDTFSFNSN PSXXXXXXXX XXXRPIF--- ---------- ----------

AauMYB1 TSDTFSFNSN PSQWFTKTG- ---R------ ---------- ----------

EgMYB2 SSPAPFP-PM DNISAPNQFD PFPTLNNRC- ---------- ----------

PtMYB4 RYQPSNAFNH EVPTAENQFC IPDVLALRH- ---------- ----------

AthMYB46 SASSLDIKDI IGSFMS--LQ EQGFVNPSL- ---------- ----------

PtrMYB3 SSSSPSI-SM HGVVTGNQFD PFTVLSNRY- ---------- ----------

PtrMYB20 SSSSSSI-SM QGMITSNRFD SFSMLNNRY- ---------- ----------

AthMYB83 SSSPFNVGPM VNSVGLNQLY DPLMISVPD- ---------- ----------

AthMYB42 ADNVNDNKIV EIDGTTTNTI DDSIITHQN- ---------- ----------

AthMYB20 MERPLENNIK NISRLEESLG DDQFM---E- ---------- ----------

AthMYB43 LEQALEKNNT SVSGDGFCID EVPLLNPHE- ---------- ----------

AmgMYB2 IQQHQQQNQQ QPVTTSLKLD QNEESDKVE- ---------- ----------

AthMYB85 EPKTTNTKYV EISVTTTEEE SSSTVTDQN- ---------- ----------

PtMYB1 SNGTVETLVS RAREPHDDIE PLQNFNMED- ---------- ----------

AthMYB26 PSPHVMTPLM FPTSREGDFK FLTTNNPNQ- ---------- ----------

AauMYB2 -NNDNKANVF VEL------- --HRDSK--- ---------- ----------

EgMYB1 -KNNAELNFM CNL------- --EESAD--- ---------- ----------

AmMYB308 ----TKHKVA VDI------- --MIKEE--- ---------- ----------

ZmMYB42 ---AAETAAQ API------- --KAEET--- ---------- ----------

ZmMYB31 ---DDKKGAV FRL------- --EDEEEEER NKATMVVGRD RQSQSHSHSH

AmMYB330 -SPSNSKNIC MPT------- --TDNNNNS- ---------- ----------

AthMYB61 ------VCVK PSISL--PPD NNSS----SP IS----GGDH VKL------A

PtMYB8 -----SIMAN PSMYR--PGP CLSS----LL YSERSLDQDL LDN------A

AauMYB3 ------LSTS SFLPN--SVC YKPS----LP VPS----DDI SMN------N

AmgMYB1 ------NMNS EFASNSISTV IAPSQTSYLG VPC----DDI CAASYAENAS

AauMYB1 ---------- ---------- ---------- ---------- ----------

EgMYB2 -----DTWEG VGFFT--FPS GIAP----VS MGD------D SSY------L

PtMYB4 -----EQVQS QNQFS--IDQ DSAT----NN LIS---HLWN SNS------T

AthMYB46 -----THIQT NNPFP--TGN MISH----PC NDD------F TPY------V

PtrMYB3 -----DVSGA ASLFD--MST CLTQ----VG MGD---GFYG DHY------G

PtrMYB20 -----DVTGA ASLFD--MST CLTQ----VG MGD---GFYG DHY------G

AthMYB83 -----NGYHQ MG--N--TVN VFSV----NG LGD-----YG NTI------L

AthMYB42 -----SSNDD YELLG--DII HNYG----DL FNI---LWTN DEP------P

AthMYB20 -----INLEY GVEDV--PLI ETES----LD LIC---SNST MSS------S

AthMYB43 -----ILIDI SSSHH--HHS NDDN----VN INT---SKFT SPS------S

AmgMYB2 -----TSYES SSTLT--ESK EDNK----VE TPP---FDDI MNS------F

AthMYB85 -----SSMDN ENHLI--DNI YDDD----EL FSY---LWSD ETT-------

PtMYB1 -----SNFNM EDSMQ--LFN VCSP----TS GIS---LSGR TEE------V

AthMYB26 -----SHHHD NNHYN--NLD ILSP----TP TIN---NHHQ PSL------S

AauMYB2 ------RGVA GERCP----- -DLN----LE LTI---SPPH QNQ------E

EgMYB1 -----VASSA RERCP----- -DLN----LE LGI---SPPS HQL------H

AmMYB308 ------NSPV QERCP----- -DLN----LD LKI---SPPC QQQ------I

ZmMYB42 ------AAVK APRCP----- -DLN----LD LCI---SPPC QHE------D

ZmMYB31 PAGEWGQGKR PLKCP----- -DLN----LD LCI---SPPC QEE------E

AmMYB330 -----SSSTD DTKCN--SST TEES----QS LIT---PPPK EEE------K

AthMYB61 APNWEFQTNN NNTSNFFDNG ---------- GFSWSIPNSS TSSSQVKPN-

PtMYB8 GTNCMNGSGA AGSAQYWDNT --------DN NNNNNVRSSS RSSSCNSST-

AauMYB3 ESQYWEATAS NHSN--RNS- ---------P FNLLPHNNTS VSYQMNMMGS

AmgMYB1 QSHFWGLTDS SKET--QIQ- ---------R LQNRTEEAKW ADYXXXXXXX

AauMYB1 ---------- ---------- ---------- ---------- ----------

EgMYB2 NLEHAKVGLL GSEFSVPPLA --------SS TTTTEENNYR SIG-CGMDG-

PtMYB4 AVSS--HESF SHAFMSPGLQ --------T- -----QGHVV KTPIKPCDQ-

AthMYB46 DGIYGVNAGV QGELYFPPLE --------C- ----EEGDWY NAN-------

PtrMYB3 ILEGNNKIGL ESDLSLPPLE --------S- -RSIEENNAV SNNRIGVKS-

PtrMYB20 ILEANNKTGL ESDLSLPPLE --------S- -RSFEENNTV SNNRIGMKS-

AthMYB83 DPISKRVSVE GDDWFIPPSE --------N- ---TNVIACS TSNNLNLQA-

AthMYB42 LVDDAS---- -WSNH----- ---------- ---------N VGIGGTAAV-

AthMYB20 TSTSSH---- -SSND----- ---------- --------SS FLKDLQFPE-

AthMYB43 SSSSTS---- -SCISSVVPG --------D- ------EFSK FFDEMEILD-

AmgMYB2 CTDEVP---- -LIEP----- ---------- --------DE ILXXXXXXX-

AthMYB85 -KDEAS---- -WSDS----- ---------- ---------N FGVGGT----

PtMYB1 DSDDSD---- -QVSKSFGNG --------SS THSQYIGRES SGVKAECGL-

AthMYB26 SCPHDNNLQW PALPDFPAST --------IS GFQETLQDYD DANKLNVFV-

AauMYB2 ----TEDQVI LKSGGRGRS- ---------- ----LCFACS LG--------

EgMYB1 -----QPEPL LRFTGRKSD- ---------- ----LCXECN LG--------

AmMYB308 ----NYHQEN LKTGGRNGSS ---------T ----LCFVCR LG--------

ZmMYB42 DGEEEDEELD LKPAFVKREA LQAGHGHGHG ----LCLGCG LG--------

ZmMYB31 --EMEEAAMR VRPA-VKREA ---------G ----LCFGCS LG--------

AmMYB330 SVPLVDLELS LGLPSQSQCN --------KS ----VSLNSS SSGFYDLFR-

AthMYB61 -HNFEEIKWS -EY------- ---------- ---LNTPFFI GSTVQS----

PtMYB8 -ANLEVNNGA AFGHFWGFGE RLDATDITSE NERKAPLFLE RADQSEYAVK

AauMYB3 QTTEEARWAD EY-------- ---------- --LHGNPIVM LAALH-----

AmgMYB1 XXXXXXXXXX XXX------- ---------- ----X-XXXX XLTPN-----

AauMYB1 ---------- ---------- ---------- ---------- ----------

EgMYB2 -KGEN----- ---SF----- ---------- -SNNNDSCFS NTTTAS----

PtMYB4 -ISWS----- ---------- ---------- ------TPLT REAAGS----

AthMYB46 ---------- ---------- ---------- --------IN N----H----

PtrMYB3 -SSN------ ---DN----- ---------- -HHFDSTCFN N-TDQR----

PtrMYB20 -SSNNN--NN -CFDN----- ---------- -TCFDNTCFN NNTDQR----

AthMYB83 -LDP------ ---------- ---------- --CFNSKNLC H--SES----

AthMYB42 -AASDKNNT- -AAEE----- ---------- -DFPERSFEK QNGESW----

AthMYB20 -FEWSDYGN- -SNND----- ---------- ---NNNGVDN IIENNM----

AthMYB43 -LKWLSSDD- -SLGD----- ---------- -DISKDGKFN NSTVDT----

AmgMYB2 -XXXXXXXX- -XXXX----- ---------- ----XXXXXX XXXXKF----

AthMYB85 --LYDHNIS- -GADA----- ---------- -DFPIWSPER INDE-K----

PtMYB1 -SGWDQMAG- -VLGD----- ---------- -PLSEWNVDL ESWAAG----

AthMYB26 -TPFNDNAKK -LLCG----- ---------- -EVLEGKVLS SSSPIS----

AauMYB2 ---LQN-SKD -CSCG----- ---------- -IVGT----- ----------

EgMYB1 ---LKN-SQN -CRCS----- ---------- -VG------- ----------

AmMYB308 ---IQN-SKD -CSC------ ---------- ---------- ----------

ZmMYB42 ---GQKGAAG -CSCS----- ---------- -NG------- ----------

ZmMYB31 ---LPR-TAD -CKCS----- ---------- -S-------- ----------

AmMYB330 -PPAKVAQRM -CVCK----- ---------- -WTLG----- ----------

AthMYB61 ---------- ---QTSQPIY IKSET----D YLA-NVS--N MTDP------

PtMYB8 WSEMLPPFSS HTQEETLPIY ITTDS----K SQD-LVSSEN INNPNHAALN

AauMYB3 ---------- --NHRAP--- ESLCN----D IKP-AS--HL VPD-------

AmgMYB1 ---------- --KQQEE--- QSQTCNMFSK DIXXXXX--X XXX-------

AauMYB1 ---------- ---------- ---------- ---------- ----------

EgMYB2 ---------- ----FKAEDD MSGFG----N -NL-QAANLR IGEWD-----

PtMYB4 ---------- ----HACNYS LGCNI----P ALV-ESETLK EKFKN-----

AthMYB46 ---------- ----LDELN- TNGSG----N -AP-EGMRPV EEFWD-----

PtrMYB3 ---------- ----FKVED- MLGLE----N -HW-QGENVR MGEWD-----

PtrMYB20 ---------- ----FKVED- MLGLE----N -HW-QGENLR MGEWD-----

AthMYB83 ---------- ----FKVGN- VLGIE----N GSW-EIENPK IGDWD-----

AthMYB42 ---------- ----MFLDYC QEFGV----E DFG-FECYHG ----------

AthMYB20 ---------- ----MSLWEI SDFSS----L DLL-LN---- ----------

AthMYB43 ---------- ----MNLWDI NDLSS----L DMF-MNEHDD GFIGN-----

AmgMYB2 ---------- ----FQLPRR PGAPR----I RVA-L----- ----------

AthMYB85 ---------- ----MFLDYC QDFGV----H DFG-F----- ----------

PtMYB1 ---------- ----LDATAA SASAW----I QQL-PDCQWN DFQGD-----

AthMYB26 ---------- ----QDHGLF LPTTY----N FQM-TSTSDH QHHHR-----

AauMYB2 ---------- ISGGTSGSGS STGGY----D FLG-LKSSVL V---------

EgMYB1 ---------- -----VIESE TSVGY----D FLG-LKASVL DYRS------

AmMYB308 ---------- ----SDGVGN ---------- ---------- ----------

ZmMYB42 ---------- ----HHFLGL RTSVL----D FRG-LEMK-- ----------

ZmMYB31 ---------- ----SSFLGL RTAML----D FRS-LEMK-- ----------

AmMYB330 ---------- ----LQKGEQ FCNCQ----S FNG-FYRYC- ----------

AthMYB61 -------WSQ NEN-----LG ----TTETSD VFSKDLQRMA VSFGQSL---

PtMYB8 SAIFPISWQQ LQNTEYPVLG DRTATMLSTP LSDPDFHRIA AVLDQI----

AauMYB3 ------TLPH HH-----HQ- ---PLQTSAI FTKDIQKLTA AFGHI-----

AmgMYB1 ------XXXX XX-----XX- ---XXXXXXX XXXXXXXXXX TDFDLVHSHF

AauMYB1 ---------- ---------- ---------- ---------- ----------

EgMYB2 ---------- ---------- --LEGLMDDL PSFPFLDF-- ----------

PtMYB4 ---------- ---------- --DAGDQINE NEIMYLPRHL L---------

AthMYB46 ---------- ---------- --LDQLMNTE VPSFYFNFKQ SI--------

PtrMYB3 ---------- ---------- --LEGLMENI SSFPFLDFQV L---------

PtrMYB20 ---------- ---------- --FEGLMENI TSFPLLDFHV E---------

AthMYB83 ---------- ---------- --LDGLIDNN SSFPFLDFQV D---------

AthMYB42 ---------- ---------- ---------- -----FGQSS MKTGHKD---

AthMYB20 ---------- ---------- ---------- ------DESS STFGLF----

AthMYB43 ---------- ---------- --GNGCS--- --RMVLDQDS WTFDLL----

AmgMYB2 ---------- ---------- ---------- ---------- ----------

AthMYB85 ---------- ---------- ---------- ---------- ----------

PtMYB1 ---------- ---------- --FEICS--- --SKSCPETL QRLGPFLDD-

AthMYB26 ---------- ---------- --VDSYINHM IIPSSSSSSP ISCGQYVIT-

AauMYB2 ---------- ---------- ---------- ---------- ----------

EgMYB1 ---------- ---------- ---------- ---------- ----------

AmMYB308 ---------- ---------- ---------- ---------- ----------

ZmMYB42 ---------- ---------- ---------- ---------- ----------

ZmMYB31 ---------- ---------- ---------- ---------- ----------

AmMYB330 ---------- ---------- ---------- ---------- ----------
