# Supplementary material for: Identification of lignin genes and regulatory sequences involved in secondary cell wall formation in Acacia auriculiformis and Acacia mangium via de novo transcriptome sequencing
Source: BMC Genomics. 2011 Jul 5;12:342. doi: 10.1186/1471-2164-12-342 (PMC3161972; doi:10.1186/1471-2164-12-342)
Supplement: Additional file 4 — Primer pairs for miRNA stem-loop sequences in A. auriculiformis. The table shows the list of primer sequences with product size and annealing temperature used in the amplification of miR160, miR172 and miR396 stem-loop sequencing in A. auriculiformis. [file 1471-2164-12-342-S4.DOC]

Primer sequences used for amplification of miR160, miR172 and miR396 stem-loop sequences in *Acacia auriculiformis*.

| miRNA stemloop | Primers | Sequence (5’-3’) | Product size/ expected size (bp) | Annealing T(°C) |
| --- | --- | --- | --- | --- |
| aau-MIR160 | miR160_F | AAGGGAATGGGATGAAGAGG | 291/243 | 58 |
|  | miR160_R | TTTGTCATTTGGGAGAGATCG |  |  |
| aau-MIR 172 | miR172_F | TATGATTGCATGGGCAGAGA | 278/287 | 58 |
|  | miR172_R | TTCACTGTGCAAAGGAGACG |  |  |
| aau-MIR 396 | miR396_F2 | TCTTCCTCTTTCTCTTTCATTATCG | 366/375 | 58 |
|  | miR396_R2 | CACATCAGTAAAGCCAATGCTCTAT |  |  |
